# Active Constituent in the Ethyl Acetate Extract Fraction of *Terminalia bellirica* Fruit Exhibits Antioxidation, Antifibrosis, and Proapoptosis Capabilities In Vitro

**DOI:** 10.1155/2019/5176090

**Published:** 2019-05-09

**Authors:** Yuxin Chen, Gao Zhou, Bingxin Ma, Jing Tong, Youwei Wang

**Affiliations:** ^1^Institute of TCM and Natural Products, School of Pharmaceutical Sciences, Wuhan University, Wuhan 430071, China; ^2^MOE Key Laboratory of Combinatorial Biosynthesis and Drug Discovery, Wuhan University, Wuhan 430072, China

## Abstract

*Terminalia bellirica* (Gaertn.) Roxb. fruit (TBF) is a widely planted traditional medicinal herb in Tibet. We aimed to determine the most active substance-enriched extract by comparing the *in vitro* antioxidant activities of different extract fractions of TBF that were subsequently extracted by petroleum ether, chloroform, ethyl acetate, and *n*-butanol after initial extraction by 95% ethanol. The main compounds of the ethyl acetate extract fraction (EF) were analyzed via HPLC-MS. Gallic acid (GA) was obtained from EF to determine *in vitro* antifibrotic activity based on the traditional usage of TBF. After HSC-T6 cells were incubated with GA, extracellular secreted levels of fibrosis-associated cytokines, such as collagen I, collagen III, TGF-*β*1, and hydroxyproline, were estimated by ELISA. Gene and protein expressions of PDGFR, CTGF, NF-*κ*B, MMP-2, TIMP-1, TIMP-2, *α*-SMA, and the Bcl-2/Bax family were determined by quantitative PCR and western blot. The proapoptotic effect of GA was further investigated by annexin V-PI and TUNEL staining. These results indicate that EF has prominent *in vitro* antioxidant activity among four extract fractions, and its main component, GA, manifests antifibrosis activity and its potential mechanism of action includes inhibition of cytokine secretion and collagen synthesis, as well as proapoptosis of HSCs.

## 1. Introduction

People living in the Qinghai-Tibet Plateau have heavy diets consisting mostly of meat and rarely eat vegetables due to the limitations brought forth by high altitude and the special geographical environment that supports the agriculture of green buckwheat and animal husbandry. These environmental features and diet as well as genetic variation are reasons why Tibetans have high risk of cardiovascular disease, hepatobiliary diseases, and oxidative stress [1]. Tibetans have used plants for generations to supplement their diet and as alternative medicine to combat oxidative stress which is an underlying cause of most diseases [2]. Literature has shown that chemicals derived from plants have capabilities to regulate the redox state in the body. The earliest application of TBF can be traced back to the 8^th^ century (Yue Wang Yao Zhen, an ancient traditional Chinese medicine book), named at that time as “Pilile.” TBF is widely planted and used in Burma, Sri Lanka, Nepal, India, and Southwest China as an important conventional medicinal plant following their own traditional Chinese medicine theory. TBF is an important medicinal herb most frequently used in the Tibetan medicine system and also used as a folk medicine by Uighurs and Mongolians. Modern pharmacological research on Pilile mainly focuses on antioxidant, antidiabetic, antihyperlipidemic, anti-HIV-1, antimalarial, antifungal, antispasmodic, and bronchodilatory properties [3–7]. However, these studies only focus on the pharmacological activities rather than the substance basis of various extracts. With technological and scientific advancements, highly sensitive analytical tools have been developed to identify and determine specific compounds in complex extracts. These tools and techniques include nuclear magnetic resonance (NMR), gas chromatography (GC), high-performance liquid chromatography (HPLC), mass spectrometry (MS), and other approaches [8]. To quickly identify the active compound, chemicals with different polarities were employed to extract TBF and the fraction with the highest antioxidant activity was selected to perform additional chemical composition analysis. Gallic acid (GA), reported as a main ingredient in TBF [9], is a natural phenolic compound which exists in various vegeles and fruits. Previous studies have shown that GA possesses a variety of pertinent biological and pharmacological activities, including antioxidant [10], anti-inflammatory [11], antifibrotic [12], and antimicrobial activities [13] and induction of cell apoptosis [14]. Nevertheless, there is no report on the antifibrotic effect of GA on HSC-T6, a rat immortal line of hepatic stellate cells (HSCs).

Liver fibrosis results from an imbalance regulation between synthesis and degradation of extracellular matrix (ECM) proteins during a scarring process [15]. HSC activation plays a key role in the development of liver fibrosis since HSCs transdifferentiate from quiescent cells present in the space of Disse to myofibroblast-like activated cells, called “activated” HSCs (aHSCs) which highly express *α*-smooth muscle actin (*α*-SMA) and ECM proteins such as collagen I and platelet-derived growth factor (PDGF) [16]. Thus, inhibiting HSC activation is a considerable way to alleviate liver fibrosis. Associated with fibrosis resolution, induction of aHSC apoptosis is another considerable strategy against liver fibrosis [17]. Therefore, suppression of HSC activation and induction of HSC apoptosis are considered two appropriate approaches for antifibrotic treatment. Our research has carried out a layer-by-layer analysis of this important traditional Tibetan medicine, from the extraction of monomeric compounds to systematically explaining the pharmacological activity of TBF and comprehensively interpreting the effects of GA on inhibiting ECM secretion and inducing cell apoptosis in HSC-T6.

In this study, we aim to compare the *in vitro* antioxidant activities of different extract fractions of TBF by a series of chemical reactions: to identify the main components of the ethyl acetate extract fraction of TBF by the HPLC-MS approach, to investigate the antifibrotic effect of GA *in vitro* by determining the expressions of ECM-associated proteins, and to study the role of GA played in inducing HSC-T6 apoptosis by fluorescent labeling.

## 2. Materials and Methods

### 2.1. Chemicals

The Folin-Ciocalteu reagent and *α*,*α*-diphenyl-*β*-picrylhydrazyl (DPPH) were purchased from Sigma (MO, USA). 2,2′-azino-bis(3-ethylbenzthiazoline-6-sulphonic acid) (ABTS) was purchased from Fluka (CA, USA). Linoleic acid was purchased from Alfa Aesar (MA, USA). Butylated hydroxytoluene (BHT) and vitamin C (Vc) were obtained from China Medicine (Group) Shanghai Chemical Reagent Corp. (China). HPLC-grade acetonitrile was purchased from Fisher Scientific (China). Gallic acid (GA), rutin, corilagin, and ellagic acid were purchased from the National Institute for the Control of Pharmaceutical and Biological Products (Beijing, China). Chebulinic acid was purchased from International Laboratory (CA, USA). All chemicals used for analysis were of analytical reagent grade. The source of cell culture reagents, ELISA kits, and PCR reagents was previously described in Chen et al. [16]. Primers used for real-time PCR were synthesized by Sangon Biotech (Shanghai, China) (Supplemental Table 1). A *β*-actin primary antibody (60008-1-Ig) was purchased from Proteintech Group Inc. (IL, USA). Primary antibodies specific to connective tissue growth factor (CTGF) (sc-14939), nuclear factor kappa-light-chain-enhancer of activated B cells (NF-*κ*B) (sc-8008), matrix metallopeptidase 2 (MMP-2) (sc-10736), tissue inhibitor of metalloproteinase 1 (TIMP-1) (sc-365905), tissue inhibitor of metalloproteinase 2 (TIMP-2) (sc-365671), B cell lymphoma 2 (Bcl-2) (sc-7382), and Bcl-2-associated X protein (Bax) (sc-7480) were obtained from Santa Cruz Biotechnology Inc. (CA, USA). Horseradish peroxidase (HRP) secondary antibodies (goat anti-mouse and goat anti-rabbit) were purchased from Abcam Inc. (MA, USA). Annexin V/PI staining was performed using an apoptosis kit from MultiSciences Biotech Co. Ltd. (Hangzhou, China). TUNEL staining was performed using in situ cell death detection Kit-POD from Roche Diagnostics Corp. (Shanghai, China).

### 2.2. Plant Materials

TBF was commercially provided by the Tibetan Traditional Medicine Pharmaceutical Factory (Lhasa City, Tibet, China). Dr. Youwei Wang (corresponding author) authenticated the voucher specimen (no. 14649), which was stored in the Traditional Chinese Medicine Specimens Museum founded by the School of Pharmaceutical Sciences, Wuhan University.

### 2.3. Preparation of Plant Extracts

200 g TBF was crushed by a grinder (powder size ≤ 0.25 mm) and subjected to heat reflux extraction using 95% ethanol. After extracting and filtering three times, the combined extract solvents were rotary evaporated at 55°C for 1 h to become concentrated. Subsequently, the residues were lyophilized by a freeze dryer (FD-1A-50, Beijing BioCool Corporation, China). The yield rate of ethanol extraction was 23.42%. Further successive extraction of the ethanol extract was performed using different solvents with increasing polarity (petroleum ether, chloroform, ethyl acetate, and *n*-butanol). The petroleum ether fraction (PF), chloroform fraction (CF), ethyl acetate fraction (EF), and n-butanol fraction (BF) were obtained, and the yield rates were 0.69%, 0.12%, 10.91%, and 3.00%, respectively.

### 2.4. DPPH Radical and ABTS Radical Scavenging Assay

Methods and calculation formulas were done without modification as previously published [18]. Briefly, in the DPPH assay, PF, CF, EF, and BF solutions were prepared at various concentrations (50, 100, 200, 400, and 800 *μ*g/mL), and BHT and Vc were used as references at the same concentration. In the ABTS assay, PF, CF, EF, and BF solutions were prepared at various concentrations (5, 10, 20, 40, and 100 *μ*g/mL), and BHT and Vc were used as references at the same concentration.

### 2.5. Superoxide Radical Scavenging Assay

The capacity of PF, CF, EF, and BF to scavenge superoxide radicals was examined by a pyrogallol autooxidation system with slight modifications [19]. Briefly, reaction mixtures dissolved in Tris-HCl buffer (4.5 mL, 50 mM, pH 8.2) containing test extracts (200 *μ*g/mL) were incubated in a water bath for 10 min at 25°C, and then 150 *μ*L of 3 mM pyrogallic acid was added. The absorbance was measured at 30 s interval at 325 nm. The autooxidation rate constant (*K*_*b*_) of pyrogallic acid was calculated by *A*_325nm_ vs. time. A control group equal to a blank group refers to no test extracts being added; BHT and Vc were used as positive controls. The *K*_*b*_ value was used to evaluate test extracts' ability to scavenge superoxide radicals.

### 2.6. Reducing Power Assay

Various concentrations (10, 40, 100, 200, and 400 *μ*g/mL) of PF, CF, EF, and BF solutions were prepared following the method described in [18]. BHT and Vc were selected as positive controls. A higher absorbance value from a plate reader at 700 nm indicates higher reducing power.

### 2.7. Antioxidant Activity in a Lipid Peroxidation (LPO) System Using *β*-Carotene Bleaching Assay

6 mg of *β*-carotene was dissolved in chloroform (20 mL). The *β*-carotene solution (4 mL) was transferred to a 500 mL round-bottomed flask and then added with 80 mg of linoleic acid and 800 mg of Tween 80. 200 mL of ddH_2_O was added and mixed well after chloroform was evaporated. 3.0 mL of the resulting linoleic acid-*β*-carotene solution was transferred to a reaction tube containing 0.2 mL of PF, CF, EF, or BF solution (400 *μ*g/mL) and incubated in a water bath at 50°C. The control was not added to PF, CF, EF, and BF sample solutions. Absorbance readings at 470 nm were recorded at 30 min intervals for 120 min. Higher absorbance equates to a higher inhibitory effect of LPO. BHT and Vc were selected as positive controls.

### 2.8. Antioxidant Activity in a LPO System Using Ferrothiocyanate (FTC) and Thiobarbituric Acid (TBA)

Antioxidative activity of each fraction was measured in a linoleic acid model system. Briefly, 0.1 mg of PF, CF, EF, and BF powder dissolved in 1 mL ethanol, respectively, was mixed with 1 mL of 2.5% linolenic acid in ethanol, 2 mL of 50 mM phosphate buffer (PBS), and 1 mL ddH_2_O. The mixtures were incubated at 40 ± 1°C in Eppendorf tubes to avoid light. Afterwards, an aliquot (0.1 mL) from the reaction mixture was mixed with 75% ethanol (9.7 mL), 30% ammonium thiocyanate (0.1 mL), and 20 mM ammonium ferrous sulfate solution (0.1 mL, formulated with 3.5% HCl). After 3 min, the absorbance was measured at 500 nm. The degree of linoleic acid oxidation was measured by the ferric thiocyanate method at 24 h intervals for 12 days.

At the last day of the FTC method, the TBA assay was employed to determine the antioxidant activity of PF, CF, EF, and BF. Briefly, 1 mL of fraction solution that was prepared the same as the FTC method above was mixed with 20% (*w*/*v*) trichloroacetic acid (TCA, 2 mL) and 0.67% (*w*/*v*) 2-thiobarbituric acid (TBA, 2 mL) and heated at 100°C for 10 min. After it was cooled to room temperature, the mixture was centrifuged at 1000 g for 10 min. The absorbance of the resulting supernatant was measured at 532 nm with a spectrophotometer. BHT was selected as references in both FTC and TBA assays.

### 2.9. High-Performance Liquid Chromatography- (HPLC-) Electrospray Ionization/Mass Spectrometry (ESI/MS) Analysis of the Main Compounds of EF

We employed HPLC to define the optimal chromatographic separation condition before performing HPLC-MS analysis, which was performed on a Shimadzu LC-20AT HPLC instrument with a UV detector using a WAT054275-Waters Symmetry C_18_ column (4.6 mm × 250 mm, 5 *μ*m). The flow rate was 1.0 mL/min. The column temperature was set at 25°C and the detection wavelength at 275 nm. The binary mobile phase comprised both solvents A (0.1% formic acid-water) and B (acetonitrile). The gradient elution started with 5% solvent B for 6 min, from 5% to 15% solvent B (6 min), constant at 15% solvent B (6 min), from 15% to 20% solvent B (7 min), constant at 20% solvent B (15 min), and finally from 20% to 5% solvent B in 5 min. The aliquots (20 *μ*L) of the standards and sample solutions were injected for HPLC analysis after the chromatographic system was equilibrated with 5% B for 15 min.

The main compounds of EF were analyzed via a HPLC system (Surveyor Plus, Thermo Fisher, USA) equipped with the same column above and an ion trap mass spectrometer (LCQ Deca XP Plus, Thermo Fisher, USA) tandem system. The binary mobile phase comprised both solvents A (0.1% formic acid-water) and B (acetonitrile). The gradient elution started with 5% solvent B for 6 min, from 5% to 15% solvent B (6 min), constant at 15% solvent B (6 min), from 15% to 20% solvent B (7 min), constant at 20% solvent B (15 min), and finally from 20% to 5% solvent B in 10 min. The flow rate was 1.0 mL/min. The injection volume was 10 *μ*L. By solvent splitting, 20% eluent was allowed to flow into the MS instrument with an ESI interface in the negative ion mode. The optimized instrumental parameters were set as follows: desolvation temperature, 230°C; source temperature, 120°C; cone voltage, 20 V; capillary voltage, 1.2 kV; desolvation gas (N_2_) flow rate, 900 L/h; auxiliary gas (He) flow rate, 40 L/h; and scan range, *m*/*z* 100–1000 amu.

### 2.10. Cell Culture

Rat HSC-T6 cells were purchased and transferred from Shuguang Hospital, Shanghai University of Traditional Chinese Medicine. Cells were thawed quickly and cultured appropriately by following the previously described method [16].

### 2.11. Antiproliferative Activity by MTT Assay

Different doses of GA (7.8, 15.6, 31.25, 62.5, 125, 250, and 500 *μ*g/mL) were prepared with DMSO in DMEM by a serial dilution method. The procedure of the MTT assay is referenced in Chen et al. [16]. 100 *μ*L/well of suspended cells was seeded in a 96-well plate and added with 100 *μ*L/well of different doses GA to meet the final concentrations (3.9, 7.8, 15.6, 31.25, 62.5, 125, and 250 *μ*g/mL). Absorbance in each well was measured at 570 nm with a microplate reader. Cell viability was calculated as follows: cell viability (%) = (OD_sample_ − OD_blank_)/(OD_control_ − OD_blank_) × 100, where OD_sample_ is the absorbance of the wells containing cells and GA or 0.1% dimethyl sulfoxide (DMSO), OD_control_ is the absorbance of the wells containing cells without GA or 0.1% DMSO, and OD_blank_ is the absorbance of the wells containing DMEM.

### 2.12. ELISA

5 × 10^5^ suspended cells/mL were seeded in 24-well plates (1 mL/well) and grown to ~80% confluence. GA was solubilized in DMSO and diluted with DMEM to achieve concentrations of 25, 50, and 100 *μ*g/mL and to make the concentration of DMSO 0.1% (*v*/*v*). In order to exclude cytotoxicity of DMSO, DMEM containing 0.1% DMSO was used as a negative control. Then, 1 mL of GA at different concentrations was added to each well to meet the final concentrations of 12.5, 25, and 50 *μ*g/mL, respectively (three technical replicates for each concentration). After a day of GA treatment, cell culture supernatants were collected in new Eppendorf tubes for subsequent ELISA experiments. Extracellular secreted levels of collagen I, collagen III, TGF-*β*1, and hydroxyproline were determined by following the manufacturer's instructions. Absorbance was read using a plate reader at 450 nm. Adherent cells were kept for further PCR and western blot analysis.

### 2.13. RNA Extraction and Real-Time PCR

Total RNA was extracted using TRIzol reagent following the instructions from the manufacturer. The real-time PCR procedure was conducted according to the method described by our previous study [16].

### 2.14. Western Blot Analysis

After the cell culture medium was aspirated, the adherent HSC-T6 cells were rinsed gently with PBS three times. Protein extraction and western blot analysis were conducted according to our previous study [16].

### 2.15. Annexin V-PI Staining for Determining HSC Apoptosis

5 × 10^5^ suspended cells/mL were seeded in six-well plates (2 mL/well) and grown to ~80% confluence. Each well contained a piece of a coverslip to let cells grow on it. Afterwards, 2 mL of GA at different concentrations was added to each well to meet the final concentrations of 12.5, 25, and 50 *μ*g/mL, respectively (three technical replicates for each concentration). After a day of GA treatment, culture medium was sucked by a vacuum, and the coverslips were then taken out and rinsed gently in PBS. The annexin V-PI staining procedure was conducted according to our previous study [16].

### 2.16. TUNEL Assay for Determining HSC Apoptosis

As described above, cells on coverslips were untreated or treated with three doses of GA for 24 h. Culture medium was sucked by a vacuum, and the coverslips were then taken out and rinsed gently in PBS and fixed in 4% paraformaldehyde for 1 h at the room temperature. After rinsing twice with PBS, the coverslips were soaked in 0.2% Triton X-100 for 5 min. Then, the coverslips were rinsed with PBS twice and the area around the samples was dried. Then, 50 *μ*L TUNEL reaction mixture was added to each sample on the coverslip and incubated in a dark humidified chamber at 37°C for 60 min. After rinsing with PBS three times and drying the area around the samples, 50 *μ*L of converter-POD was added to each coverslip and the coverslips were incubated in a dark humidified chamber at 37°C for 60 min. Then, the coverslips were rinsed with PBS three times and mounted on glass slices with glycerol. The coverslips were examined using a fluorescence microscope (Nikon Ti-E, Japan) with an excitation wavelength in the range of 450-500 nm and a detection wavelength in the range of 515-565 nm.

### 2.17. Statistical Analysis

All data are mean ± SD represented by the error bars. Means were considered significantly different when *p* < 0.05. Statistical differences were analyzed by one-way ANOVA and the LSD test (IBM SPSS Statistics 20.0).

## 3. Results and Discussion

### 3.1. Antioxidant Activities of Four Extract Fractions of TBF in the Free Radical Scavenging and Lipid Peroxidation System

**Figure 1:**
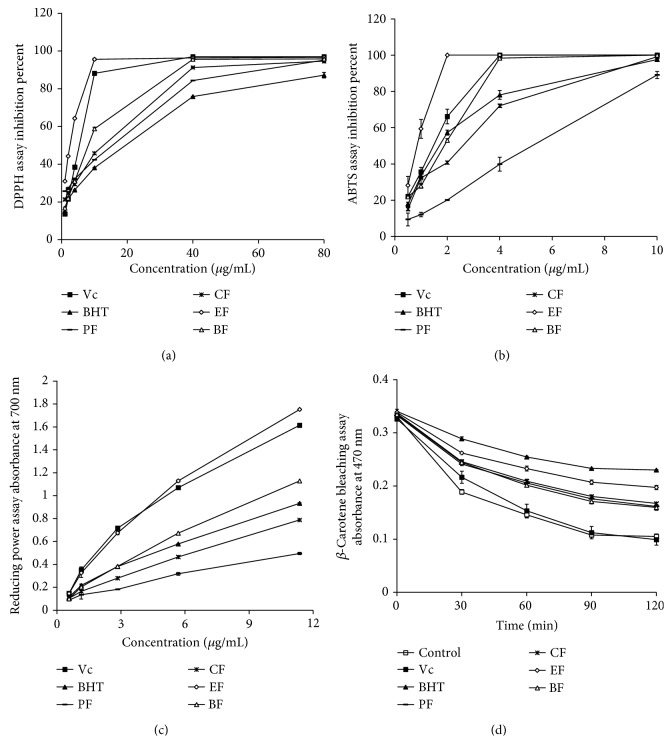
Antioxidant activity of fractions of TBF was determined by (a) DPPH radical scavenging assay, (b) ABTS radical scavenging assay, (c) reducing power assay, and (d) *β*-carotene bleaching assay. PF: petroleum ether fraction; CF: chloroform fraction; EF: ethyl acetate fraction; BF: *n*-butanol fraction; BHT: butylated hydroxytoluene; Vc: vitamin C.

The DPPH radical was used to evaluate the scavenging activity of antioxidants by accepting hydrogen atom or electron donation resulting in a bleaching of a purple-colored methanol solution. As shown in Figure 1(a), the different concentrations of PF, CF, EF, and BF showed concentration-dependent radical scavenging activities with IC_50_ values of 13.88 ± 0.27 *μ*g/mL, 11.04 ± 0.85 *μ*g/mL, 1.10 ± 0.08 *μ*g/mL, and 6.78 ± 0.23 *μ*g/mL, while the IC_50_ value of Vc and BHT was 6.68 ± 1.14 *μ*g/mL and 19.36 ± 0.24 *μ*g/mL, respectively. This showed that the ability of EF to scavenge DPPH free radical was superior to that of Vc and BHT. BF has a comparable DPPH free radical scavenging ability with Vc while PF and CF are valued between the Vc and BHT. Naik et al. reported that the IC_50_ value of the aqueous extract of TBF for DPPH free radical scavenging was 10.00 *μ*g/mL [20]. Hazra et al. reported that the IC_50_ value of the 70% methanol extract of TBF for DPPH free radical scavenging was 1.45 ± 0.02 *μ*g/mL [21]. The IC_50_ value of the ethyl acetate extract for DPPH free radical scavenging was 1.40 *μ*g/mL. All the IC_50_ values they reported for DPPH free radical scavenging were higher than what we reported (1.10 ± 0.08 *μ*g/mL). This can be explained by the fact that different solvent extractions have different effects on concentrating active compounds; the extraction with ethyl acetate after ethanol extraction is considered a better way to concentrate active compounds in TBF. In short, the TBF purchased from Tibet has excellent antioxidation effects after undergoing extraction by our method. Such exceptional antioxidation effect has never been reported by other researchers.

ABTS is also a widely accepted free radical compound to estimate the scavenging capacity of antioxidants. In Figure 1(b), EF (IC_50_ = 0.82 ± 0.09 *μ*g/mL) and BF (IC_50_ = 1.89 ± 0.04 *μ*g/mL) were found to be very effective free radical scavengers, while PF (IC_50_ = 5.30 ± 0.53 *μ*g/mL) and CF (IC_50_ = 2.03 ± 0.10 *μ*g/mL) showed weaker scavenging abilities relative to Vc (IC_50_ = 1.38 ± 0.15 *μ*g/mL) and BHT (IC_50_ = 1.63 ± 0.03 *μ*g/mL). EF was valued as having the best ABTS free radical scavenging ability among the four extract fractions, even stronger than Vc and BHT. ABTS radical cation instantaneously formed once potassium persulfate was added to an ABTS solution. Phani Kumar et al. reported that the ethyl acetate fraction of the ethanolic extract of *Terminalia arjuna* skin had an IC_50_ value of 25 ± 1.2 *μ*g/mL for ABTS free radical scavenging [22], while our EF had IC_50_ = 0.82 ± 0.09 *μ*g/mL, proving that EF is significantly better at scavenging ABTS free radicals.

The superoxide anion radical (^·^O_2_^−^) is a ubiquitously generated free radical in vivo, which can be produced from pyrogallic acid spontaneous oxidation under alkaline conditions, and also disappears rapidly by reacting with the hydrodioxyl radical under acidic conditions. The fractions can hinder the autooxidation reaction of pyrogallic acid in this in vitro system due to their abilities to scavenge ^·^O_2_^−^ radicals (Table 1). The lower *K*_*b*_ value (×10^−4^ A/s) indicates better scavenging of ^·^O_2_^−^ radicals. As indicated in Table 1, the degree of ^·^O_2_^−^ radical scavenging of CF, EF, and BF was higher than that of BHT, while PF has an equivalent scavenging ability with BHT. However, Vc has the strongest ability to scavenge ^·^O_2_^−^ compared to all other fractions. EF showed the best scavenging ability among all the four fractions, and we compared its *K*_*b*_ value with that of other extracts investigated in our lab. Huang et al. reported that the *K*_*b*_ value of the superoxide anion radical scavenging by the 70% methanol extract of *Halenia elliptica* was 10.87 ± 0.02 (×10^−4^ A/s) [19]. Zhou et al. reported that the *K*_*b*_ value of the superoxide anion radical scavenging by the 70% ethanol extract of *Meconopsis integrifolia* was 3.93 ± 0.30 (×10^−4^ A/s) [23]. These studies indicate that under the same experimental method, the efficiency of EF scavenging superoxide anion radicals is higher than that of the 70% methanol extract of *Halenia elliptica* but slightly less than that of the 70% ethanol extract of *Meconopsis integrifolia*.

The reducing power of a substance is associated with its potential antioxidant activity. The Fe^3+^/ferricyanide complex reduces to the ferrous form (Fe^2+^) when antioxidants are present, and Fe^2+^ concentration can be monitored by measuring the formation of Perl's Prussian blue at 700 nm [24]. A higher absorbance value indicated stronger reducing power by the samples, as shown in Figure 1(c); the order of reducing power by the extracts is EF > Vc > BF > BHT > CF > PF. EF showed significantly greater reducing power than Vc. Hazra et al. reported that the 70% methanol extract of TBF has almost no change in reducing power over the entire concentration range (0.0-1.0 mg/mL) [21], a conclusion different from our results. Our fractions showed rapidly rising reducing power in this concentration range.

Because of the coupled oxidation of *β*-carotene and linoleic acid, *β*-carotene discolors rapidly in the absence of an antioxidant to scavenge free radicals. The linoleic acid free radical, formed by drawing a hydrogen atom from its diallylic ethylene groups, attacks the highly unsaturated *β*-carotene molecules [25]. Consequently, *β*-carotene becomes oxidized and loses its chromophore and characteristic orange color. The antioxidants present in the system can impede the extent of *β*-carotene bleaching by neutralizing the linoleic acid free radical. As a result, the absorbance decreases rapidly in samples lacking sufficient antioxidants, while samples with antioxidants have their orange color retained.  Figure 1(d) shows that the absorbance of the control and Vc group fell significantly as time increased. The BHT presented excellent antioxidant ability, of which the LPO inhibition value was 51.2%. The LPO inhibition value of EF, CF, PF, and BF was 37.5%, 25.1%, 24.2%, and 22.4%, respectively, which indicated that EF has the best ability to fade *β*-carotene among the four fractions. From the results of the *β*-carotene bleaching assay, we concluded that the positive control Vc was not suitable for the lipid peroxidation system, so only BHT was used as a positive control in the subsequent anti-LPO experiments.

**Figure 2:**
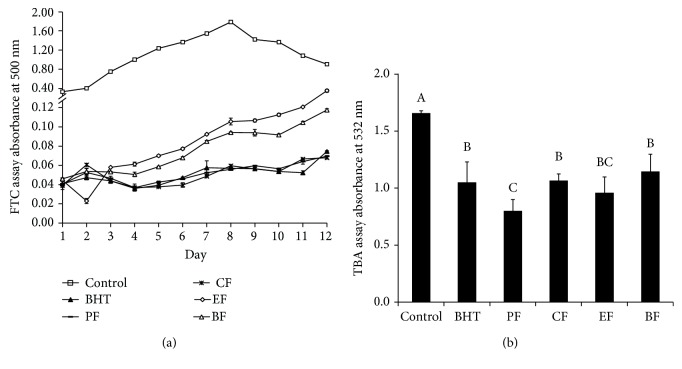
Antilipid peroxidation activity of fractions of TBF was examined by (a) FTC and (b) TBA assay. PF: petroleum ether fraction; CF: chloroform fraction; EF: ethyl acetate fraction; BF: *n*-butanol fraction; BHT: butylated hydroxytoluene; Vc: vitamin C. Different letters differed significantly (*p* < 0.05).

The FTC method was used to measure the amount of peroxide produced during the linoleic acid oxidation. Peroxides react with Fe^2+^ to form Fe^3+^, which has a maximum absorbance at 500 nm with SCN^−^. In our assay, the formation of peroxides and oxidation of Fe^2+^ were quenched on day 8 due to shortage of linoleic acid. Therefore, the absorbance at 500 nm begins reducing after day 8. Accordingly, the oxidation of linoleic acid would be slow in the presence of antioxidants. The effect of fractions on preventing the peroxidation of linoleic acid is shown in Figure 2(a). We can see that PF, CF, and BHT showed lower absorbance values indicating better resistance abilities to linoleic acid oxidation. Interestingly, PF and CF exhibited better resistance abilities to linoleic acid oxidation than EF and BF, whereas in the previous free radical scavenging experiments, both PF and CF activities were less than the activities of EF and BF. This suggests that the compounds contained in these fractions exhibit different patterns of antioxidant activity. The compounds contained in PF and CF are more suitable for combating oxidation in the LPO system while the compounds contained in EF and BF are more suitable for scavenging free radicals.

The TBA method is a measure of the final products of the lipid peroxidation assay, including small molecules such as aldehydes, ketones, acids, and hydrocarbons, which have maximum absorption peaks between 532 and 535 nm. The lower the absorbance value, the less the end products of LPO.  Figure 2(b) shows that BHT, PF, CF, EF, and BF all inhibited lipid peroxide production significantly compared with the control group, which indicated that PF, CF, EF, and BF can effectively remove the TBA substrate (MDA, etc.) in the postoxidation system. CF, EF, BF, and BHT were equally effective, and there was no significant difference between them. Unexpectedly, PF showed the best activity against lipid peroxide production compared to other extracts and was significantly better than BHT (*p* < 0.05). This may be due to the fact that PF performed best in the process of inhibiting lipid peroxidation in the early stage (FTC assay), so that no excessive MDA and other substances were produced in this system. Sarin et al. reported that oleic acid glyceride (61.5%) dominates in *Terminalia bellirica* Roxb. seed oil [26]. Low-density lipoproteins rich in oleic acid have been shown to combat changes in oxidative stress [27]. The presence of unsaturated fatty acids enriched in PF and CF during the extraction process may contribute to the performance of PF in FTC and TBA experiments.

In summary, EF has relatively prominent in vitro antioxidant activity in both free radical scavenging and antilipid peroxidation systems among the four fractions. It is worth exploring the molecular basis of this important extract regarding whether EF can achieve extremely excellent antioxidant effects.

### 3.2. HPLC-ESI/MS Analysis for EF

**Figure 3:**
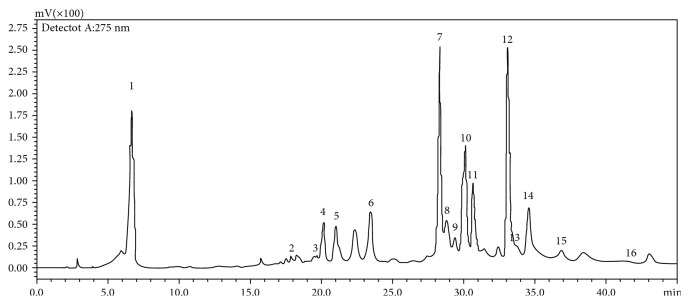
HPLC chromatogram of substances in the ethyl acetate extract fraction of TBF at 275 nm. Numbers 1-16 represent substances that can be recognized by a mass spectrometer.

The liquid chromatogram of the EF was obtained by Shimadzu HPLC at 275 nm (Figure 3). The total ion current chromatogram obtained by the Thermo Fisher HPLC-ESI/MS system is shown in Supplemental Figure 1, and the results of tentative identification are shown in Table 2; also, the chemical structures of these compounds are shown in Supplemental Figure 3. Gallic acid (1), methyl neochebulanin (2), 3′-O-methyl-4-O-(3^″^,4^″^-di-O-galloyl-*α*-L-rhamnopyranosyl) ellagic acid (3), chebulanin (4), corilagin (5), 3,4,6-tri-O-galloyl-*β*-D-Glc (6), chebulagic acid (7), methyl neochebulagate (8), 1,3,4,6-tetra-O-galloyl-*β*-D-Glc (9), ellagic acid (11), chebulinic acid (12), methyl neochebulinate (13), 1,2,3,4,6-penta-O-galloyl-*β*-D-Glc (14), and 3′-O-methyl-4-O-(*β*-D-xylopyranosyl) ellagic acid (16) were identified based on the molecular weight given by MS and the previous report [9]. MS spectra for each of 16 compounds can be found in Supplemental Figure 2.

The 14 compounds identified by HPLC-MS can be classified into five categories: (1) gallic acid and simple gallate esters (1, 6, 9, and 14), (2) chebulic acid and chebulic ellagitannins (2, 4, 7, 8, 12, and 13), (3) nonchebulic ellagitannins (5), (4) ellagic acid and derivatives (11), and (5) ellagic glycosides (3 and 16). This shows that EF is enriched in a large amount of phenolic compounds. Many studies have reported that phenolic compounds play an indispensable role in antioxidant activity [28, 29]. There are two compounds still unknown (10 and 15), but their MS spectra show categorical molecular ion peaks and high relative abundance (Supplemental Figure 2). Based on our preliminary report, it requires further phytochemical research to identify them.

The enrichment effect of ethyl acetate on gallic acid and simple gallic acid ester compounds is well known. Gallic acid (GA) and ellagic acid in mango kernel and longan seed have been shown to have good activity in scavenging ABTS free radicals [30]. EF may have strong antioxidant capacities due to its high content of GA. The traditional use of TBF is to treat hepatobiliary diseases, so we decided to study the ability of GA to fight against liver fibrosis.

### 3.3. The Effect of GA on Inhibiting HSC-T6 Cell Proliferation and Synthesis of Fibrotic Cytokines

The antiproliferative activity of GA in HSC-T6 cells was indicated by the MTT assay. The IC_50_ value was determined as 41.39 *μ*g/mL after coincubating with GA for 24 h (Supplemental Figure 4). Thus, 12.5, 25, and 50 *μ*g/mL were selected as low, medium, and high doses, respectively, of GA for further analysis.

**Figure 4:**
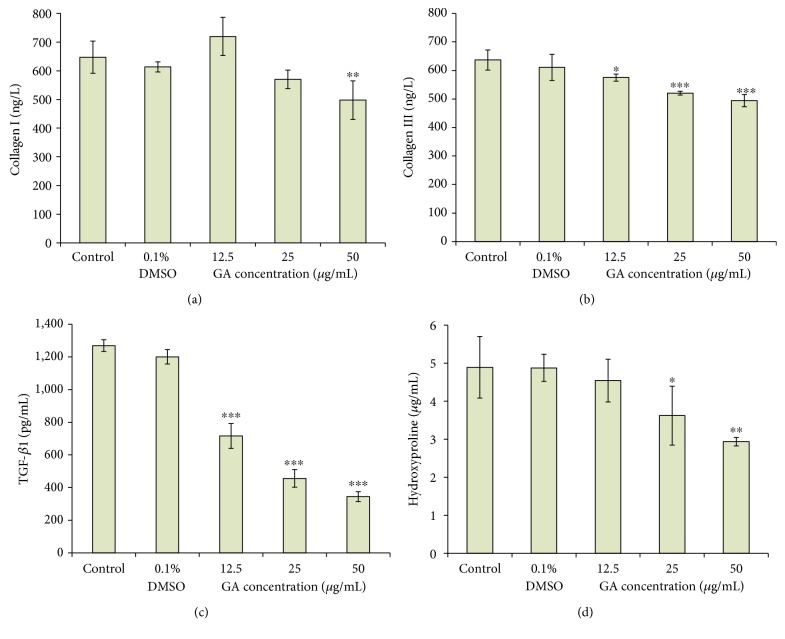
Effect of GA on (a) collagen I synthesis, (b) collagen III synthesis, (c) TGF-*β*1 level, and (d) hydroxyproline content in HSC-T6 cells. Cells were treated with GA at various concentrations (12.5, 25, and 50 *μ*g/mL) for 24 h and then measured using assay kits. Cells were treated with 0.1% DMSO as a vehicle control. ^∗^ *p* < 0.05, ^∗∗^ *p* < 0.01, and ^∗∗∗^ *p* < 0.001.

After medium-dose (50 *μ*g/mL) GA treatment, the type I collagen content in the ECM was significantly reduced compared to the control group (Figure 4(a)). The content of type III collagen was significantly decreased in low, medium, and high concentrations of GA (Figure 4(b)). These two experimental results indicate that GA can effectively reduce collagen deposition in the ECM. After the cells were treated with 50 *μ*g/mL of GA, the content of type I collagen and type III collagen in the ECM was reduced by 23.15% and 22.37%, respectively. The TGF-*β*1 content was significantly reduced (*p* < 0.001) by GA in a dose-dependent manner (Figure 4(c)). Further analysis showed that the low, medium, and high concentrations of GA decreased TGF-*β*1 protein secreted to the ECM by 43.55%, 64.02%, and 72.83%, respectively. After treatment with 25 *μ*g/mL and 50 *μ*g/mL of GA, the content of hydroxyproline in the ECM was reduced by 25.97% and 39.99%, respectively (Figure 4(d)), and was significantly different from that of the control group (*p* < 0.05, *p* < 0.01). In Figures 4(a)–4(d), there was no significant difference between the control group and the negative control group, indicating that the effect of 0.1% DMSO on cell growth can be omitted. Patel and Goyal observed that GA could effectively decrease collagen and protein content in diabetic rats [31]. There is no standard treatment for liver fibrosis. Among those inflammatory cytokines involved in liver fibrosis, TGF-*β*1 appears to be the most important one. In our previous study, we proved that GA in medium and high doses could significantly reduce TGF-*β*1 and hydroxyproline in rats with liver fibrosis [32], which is consistent with the results found in this study. Cheng et al. provided a prospect that silencing of TGF-*β*1 by siRNA and shRNA might be an efficient approach against liver fibrosis [33].

### 3.4. Effects of GA on Gene and Protein Expressions of Fibrotic Cytokines and Proapoptotic Proteins

**Figure 5:**
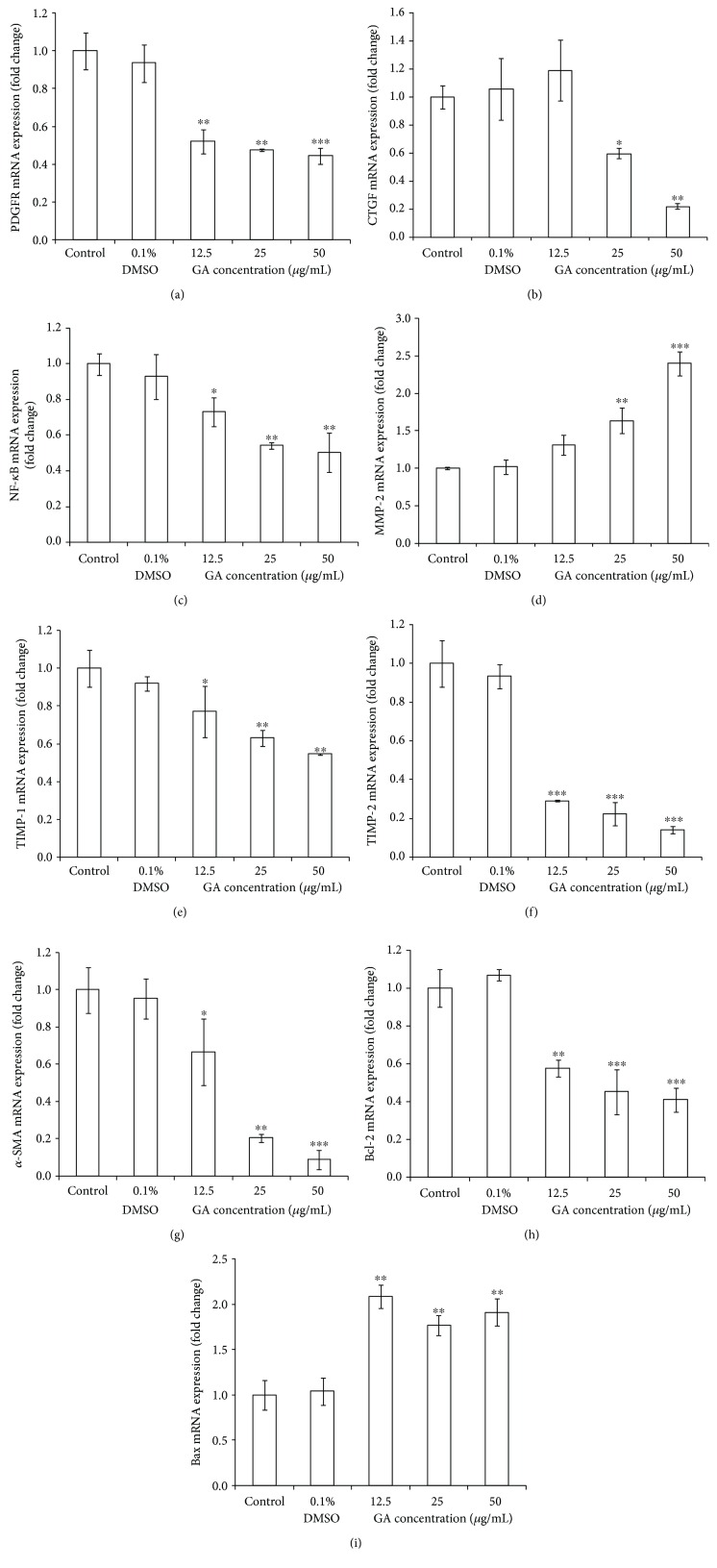
Effect of GA on (a) PDGFR, (b) CTGF, (c) NF-*κ*B, (d) MMP-2, (e) TIMP-1, (f) TIMP-2, (g) *α*-SMA, (h) Bcl-2, and (i) Bax gene expression in HSC-T6 cells. Cells were treated with GA at various concentrations (12.5, 25, and 50 *μ*g/mL) for 24 h. Cells were treated with 0.1% DMSO as a vehicle control. Real-time PCR was performed to detect mRNA levels of target genes. Results are presented as relative changes normalized to GAPDH. ^∗^ *p* < 0.05, ^∗∗^ *p* < 0.01, and ^∗∗∗^ *p* < 0.001.

**Figure 6:**
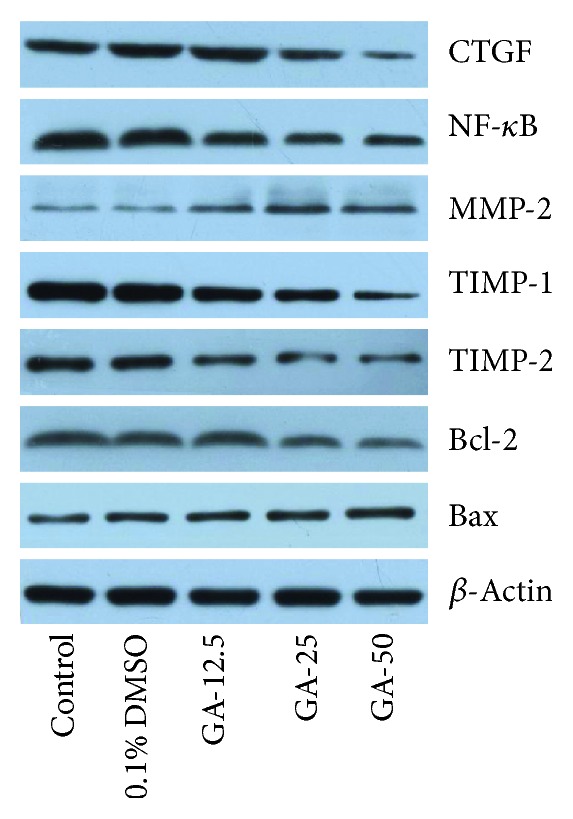
Effect of GA on CTGF, NF-*κ*B, MMP-2, TIMP-1, TIMP-2, Bcl-2, and Bax protein expression in HSC-T6 cells. After incubation with GA at various concentrations (12.5, 25, and 50 *μ*g/mL) for 24 h, protein levels were examined in HSC-T6 cells by western blotting.

After the cells were treated with various doses of GA for 24 h, we evaluated the expression of platelet-derived growth factor receptor (PDGFR) mRNA to analyze the response of cells to GA. The decrease in PDGFR gene expression caused the decrease in the cell response to PDGF, and GA at each concentration played a significant role (*p* < 0.01) in decreasing PDGFR gene expression (Figure 5(a)). There was some increase in the expression of CTGF after treatment with 12.5 *μ*g/mL of GA (Figure 5(b)), but alteration in the expression level was not statistically significant. Significant decreases were observed after treatment with medium and high doses of GA. In particular, high dose of GA significantly decreased the expression level of CTGF by 5-fold. Each concentration of GA was effective in inhibiting the expression of NF-*κ*B mRNA (Figure 5(c)). We also observed a gradual increase in MMP-2 gene expression after GA treatment at three doses, and the highest concentration of GA increased the gene expression of MMP-2 by 2.4-fold (Figure 5(d)). Compared to the control group, the mRNA expression of TIMP-1 or TIMP-2 was significantly lower in each GA-treated group (12.5, 25, and 50 *μ*g/mL), and the effect of GA on the mRNA level of TIMP-2 was more pronounced (Figures 5(e) and 5(f)). The mRNA expressions of *α*-SMA after treatment with various concentrations of GA were significantly downregulated (*p* < 0.05), showing a significant dose-dependent effect (Figure 5(g)) with the effect of 50 *μ*g/mL GA being the strongest.  Figure 5(h) shows that the three GA treatment groups can significantly reduce (*p* < 0.01) the mRNA expression of Bcl-2 while Figure 5(i) shows that they can significantly increase (*p* < 0.01) the mRNA expression of Bax. Interestingly, in Figure 5(i), 12.5 *μ*g/mL GA treatment resulted in the highest mRNA expression of Bax while 50 *μ*g/mL GA showed the second highest effect. However, there were no significant differences between the three treatment groups. All of the above experiments showed that 0.1% DMSO treatment has no effect on expressions of the target genes. In summary, we evaluated the effect of GA on reducing the accumulated ECM in HSC-T6 cells by monitoring the gene expressions of fibrotic cytokines and proapoptotic proteins. The protein expression of CTGF, NF-*κ*B, MMP-2, TIMP-1, TIMP-2, Bcl-2, and Bax has similar alteration trends to those of their mRNA expressions as shown in Figure 6. In the present study, we examined liver fibrosis by using a model cell, HSC-T6. Numerous attempts over many years were made to develop therapy to cure or mitigate liver fibrosis. It is recognized that HSC activation plays a pivotal role in the development of liver fibrosis and that the PDGF-PDGFR interaction plays a central role in HSC activation [34]. Ding et al. have reported that propranolol inhibited PDGF-BB–induced hepatic stellate cell activation through PDGFR/Akt phosphorylation [35]. In normal adult fibroblasts, TGF-*β* induces the expression of CTGF that independently promotes fibroblast proliferation and matrix deposition [36]. CTGF has been reported to stimulate a two- to three-fold increase in pro*α*1(I) collagen and fibronectin synthesis by both dermal and lung fibroblasts in culture and promote significant matrix remodeling of fibroblast-populated three-dimensional collagen lattices [37]. NF-*κ*B was detected as a protein that could complex to a 10 bp site in the *κ* light chain enhancer, called *κ*B [38]. When unstimulated, NF-*κ*B is present in the cell in its inactive form, bound to an inhibitory protein called I*κ*B [39]. Many factors in cells can activate NF-*κ*B, such as growth factors, bacteria, viruses, UV radiation, and oxidative stress [40]. The literature reports that NF-*κ*B is present in activated fibrotic cells and is closely related to the progression of fibrosis [41, 42]. TGF-*β*1 was proven to regulate the expressions of matrix metalloproteinases (MMPs) and tissue inhibitors of metalloproteinase (TIMPs), which promote ECM degradation and ECM synthesis, respectively [43]. Shikonin, a natural product extracted from dried roots of *Lithospermum erythrorhizon*, has been proven to significantly reduce TIMP-1 expression and improve MMP-2 expression of either mRNA expression or protein level in a CCl_4_-induced or bile duct ligation-induced liver fibrosis model [44]. *α*-SMA is an actin isoform and a specific marker for smooth muscle cell differentiation, which has been used to identify aHSCs to show a myofibroblastic phenotype [45]. Lu et al. have reported that the water extract of *Phyllanthus emblica* L. fruits and its major compound ellagic acid were able to markedly reduce protein levels of *α*-SMA in HSC-T6 cells [46]. Driving activated HSCs into apoptosis may be another way to resolve fibrosis as many studies have reported the link between apoptosis and fibrosis [47]. BCL-B, a member of the BCL-2 family, has been proven to inhibit both apoptosis and mitophagy in human HSCs [48]. Similarly, our result shows that GA promotes HSC apoptosis by suppressing Bcl-2 expression. Hsieh et al. demonstrated that 100-150 *μ*M of GA treatment caused an imbalance between Bcl-2 and Bax, which induced the apoptosis of hypertrophic scar fibroblasts [49]. Their results and the results in this paper together confirm that GA plays a role in proapoptosis in fibroblasts. In summary, we observe that the expressions of cytokines or apoptotic proteins that determine fibrogenesis were suppressed/elevated by various concentrations of GA treatment on HSC-T6, suggesting that GA has a potential ability to reverse liver fibrosis.

### 3.5. Detection of GA-Induced HSC-T6 Apoptosis by Fluorescent Staining

**Figure 7:**
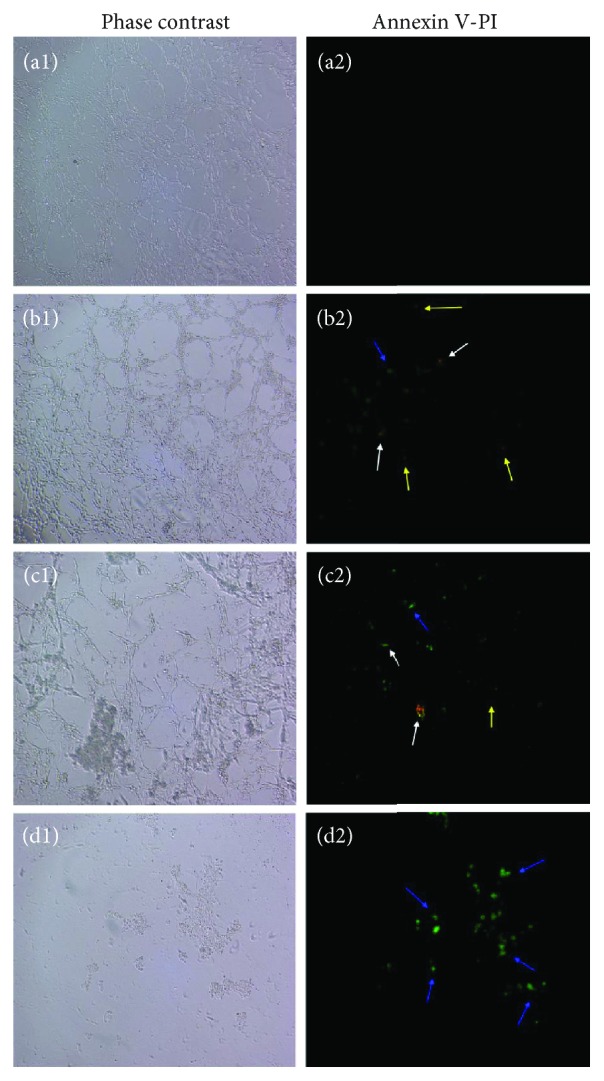
Effect of GA on HSC-T6 apoptosis was measured via annexin V-PI staining (200x). (a1, a2) Control. (b1, b2) Cells were incubated with 12.5 *μ*g/mL GA. (c1, c2) Cells were incubated with 25 *μ*g/mL GA. (d1, d2) Cells were incubated with 50 *μ*g/mL GA. Cells stained only with green by annexin V are in the early stage of apoptosis (blue arrow), cells stained only in red by PI are damaged cells (yellow arrow), and cells stained in both green and red are in advanced apoptosis or in necrosis (white arrow).

**Figure 8:**
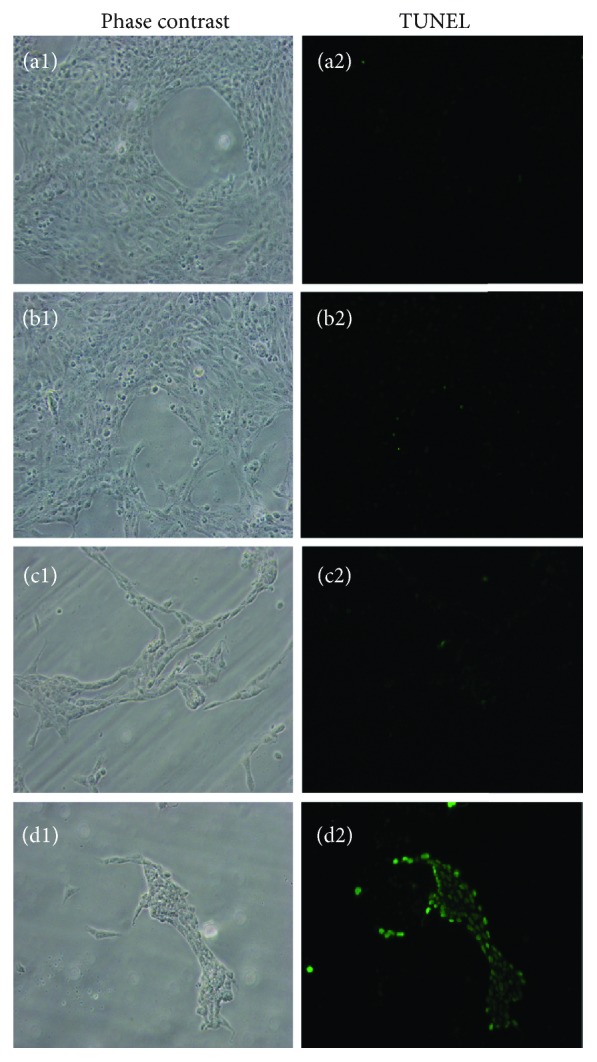
Effect of GA on HSC-T6 apoptosis was evaluated through the TUNEL assay (400x). The morphology of HSCs was investigated under a fluorescence microscope. TUNEL-positive cells (green light) were considered apoptotic cells. (a1, a2) Control. (b1, b2) Cells were incubated with 12.5 *μ*g/mL GA. (c1, c2) Cells were incubated with 25 *μ*g/mL GA. (d1, d2) Cells were incubated with 50 *μ*g/mL GA.

Since GA simultaneously upregulated proapoptosis genes and downregulated antiapoptosis genes, we next evaluated the ability of GA to promote apoptosis of hepatocytes using annexin V-PI and TUNEL assay staining on HSC-T6 cells. After treatment with 12.5, 25, and 50 *μ*g/mL of GA for 24 h, cells were stained with annexin-V/FITC and PI. The results indicate that the amount of apoptotic HSC-T6 cells that were cocultured with GA significantly increased in a dose-dependent manner compared to the untreated cells (Figures 7(a2)–7(d2)). The fluorescence microscope was switched to normal light mode to visualize cell morphology (Figures 7(a1)-7(d1)). Annexin V is a phospholipid binding protein with high affinity of phosphatidylserine. A green fluorescence signal which is emitted by FITC can be detected when cells are in an initial stage of apoptosis (blue arrow in the figure), suggesting that phosphatidylserine (PS) externalization is occurring; propidium iodide (PI) stains the nucleus and its fluorescence signal is red, which can be detected when the cell membrane loses its integrity (yellow arrow in the picture). Cells with both green and red fluorescence signals detected simultaneously may be undergoing advanced stages of apoptosis or cell necrosis (white arrows in the figure). We can see that the number of cells under light microscopy is drastically reduced when they were treated with 50 *μ*g/mL of GA. This is because the highest concentration of GA used is greater than the IC_50_ value we obtained from the MTT assay. Cell viability was only 30% when the cells were undergoing 50 *μ*g/mL GA treatment. Wang et al. observed similar fluorescent images when they tested whether curcumin had the ability to protect against thioacetamide-induced hepatic fibrosis by inducing apoptosis of damaged hepatocytes [50]. The TUNEL method confirmed that HSC-T6 cells cocultured with each concentration of GA for 24 hours were able to generate genomic DNA fragments (Figures 8(b2)–8(d2)). The green fluorescence signal indicated apoptotic cells; as the concentration of GA increased, the number of total cells decreased and the number of apoptotic cells increased. Almost no apoptotic cells were observed in the control group (Figure 8(a2)). Cell morphology is visible under light microscopy with phase contrast (Figures 8(a1)-8(d1)). Hsieh et al. observed similar fluorescent images and dose effects of GA to induce apoptosis in a similar manner as our results instead in hypertrophic scar fibroblasts [49]. Taken together, our data strongly suggest that GA can inhibit the proliferation of HSC-T6 cells by initialing apoptosis.

In summary, our study first provides evidences that EF of TBF possesses excellent antioxidant capacity, especially in its outstanding free radical scavenging ability; one out of sixteen active constituents of EF, GA, effectively reduced the accumulated ECM in cultured HSC-T6 cells. GA mediates gene expression and protein levels of the Bcl-2/Bax family to induce HSC-T6 cell apoptosis. All of these can be considered potential antifibrotic mechanisms of GA.

## Figures and Tables

**Table 1:** Inhibition of pyrogallic acid autooxidation by fractions of TBF.

Sample	Control	PF	CF	EF	BF	BHT	Vc
*K*_*b*_ value (×10^−4^)	7.44 ± 0.20^a^	6.79 ± 0.08^b^	6.10 ± 0.23^c^	5.21 ± 0.21^d^	5.84 ± 0.09^c^	6.55 ± 0.22^b^	0.11 ± 0.07^e^

TBF: *Terminalia bellirica* (Gaertn.) Roxb. fruit; PF: petroleum ether fraction; CF: chloroform fraction; EF: ethyl acetate fraction; BF: *n*-butanol fraction; BHT: butylated hydroxytoluene; Vc: vitamin C. Different letters differed significantly (*p* < 0.05).

**Table 2:** Retention times and MS parameters for the 16 putative compounds in EF.

Peak	*T*_R_ (min)	*T*_R_ (min) on total ion current chromatogram	[M-H]^−^ (*m*/*z*)	Tentative identification
1	6.702	12.52	169.47	Gallic acid
2	17.906	21.76	683.06	Methyl neochebulanin
3	19.482	23.75	764.70	3′-O-Methyl-4-O-(3^″^,4^″^-di-O-galloyl-*α*-L-rhamnopyranosyl) ellagic acid
4	20.153	23.77	651.09	Chebulanin
5	21.06	25.21	633.31	Corilagin
6	23.528	28.01	635.39	3,4,6-Tri-O-galloyl-*β*-D-Glc
7	28.366	31.47	953.45	Chebulagic acid
8	28.824	32.31	985.12	Methyl neochebulagate
9	29.402	33.74	787.21	1,3,4,6-Tetra-O-galloyl-*β*-D-Glc
10	30.138	34.65	610.48	Unknown
11	30.669	35.63	301.47	Ellagic acid
12	33.135	37.82	955.12	Chebulinic acid
13	33.621	37.92	987.10	Methyl neochebulinate
14	34.610	40.16	939.15	1,2,3,4,6-Penta-O-galloyl-*β*-D-Glc
15	36.914	41.23	723.67	Unknown
16	41.528	45.14	447.34	3′-O-Methyl-4-O-(*β*-D-xylopyranosyl) ellagic acid

## Data Availability

The data used to support the findings of this study are available from the corresponding author upon request.

## Supplementary Materials

Supplementary Materials Table S1: primer sequences used for real time-PCR analysis. Supplemental Figure 1: the total ion current (TIC) output from the ESI-MS in negative mode. Supplemental Figure 2: MS spectra of the 16 putative compounds in EF. The number of each spectrum corresponds to the peak number in Table 2, Figure 1, Supplemental Figure 1, and Supplemental Figure 3. Supplemental Figure 3: structures of the 14 phenolic compounds identified from EF based on the HPLC-MS data and the known structures of reference compounds. Compound numbers, chemical formula, and the exact mass are presented. Supplemental Figure 4: effect of GA on HSC-T6 cell viability. The viability rate (%) of HSC-T6 cells decreased with increased GA concentration.
