# Supplementary material for: Active Constituent in the Ethyl Acetate Extract Fraction of Terminalia bellirica Fruit Exhibits Antioxidation, Antifibrosis, and Proapoptosis Capabilities In Vitro
Source: Oxid Med Cell Longev. 2019 May 9;2019:5176090. doi: 10.1155/2019/5176090 (PMC6532289; doi:10.1155/2019/5176090)
Supplement: Supplementary Materials — Table S1: primer sequences used for real time-PCR analysis. Supplemental Figure 1: the total ion current (TIC) output from the ESI-MS in negative mode. Supplemental Figure 2: MS spectra of the 16 putative compounds in EF. The number of each spectrum corresponds to the peak number in Table 2, Figure 1, Supplemental Figure 1, and Supplemental Figure 3. Supplemental Figure 3: structures of the 14 phenolic compounds identified from EF based on the HPLC-MS data and the known structures of reference compounds. Compound numbers, chemical formula, and the exact mass are presented. Supplemental Figure 4: effect of GA on HSC-T6 cell viability. The viability rate (%) of HSC-T6 cells decreased with increased GA concentration. [file 5176090.f1.pdf]

Table S1. Primer sequences used for real time-PCR analysis

Gene	Direction	Sequence
GAPDH	Forward	GGCATCGTGGAAGGGCTCAT
	Reverse	AGGGATGATGTTCTGGGCTGC
PDGFR	Forward	CATTGGGGACAGGGAAGTGGAC
	Reverse	CCTGATGGTGATGCTCTCGC
CTGF	Forward	CTGAAAGAATAGCTGGCTTCA
	Reverse	CAGGTACTAGCTGAGGTCAT
NF-κB	Forward	AACACTGCCGAGCTCAAGAT
	Reverse	CATCGGCTTGAGAAAAGGAG
MMP-2	Forward	CTTGCTGGTGGCCACATTC
	Reverse	CTCATTCCCTGCGAAGAACAC
TIMP-1	Forward	GCCTACACCCCAGCCAT
	Reverse	ATGCCAGGGAACCAGGAAGC
TIMP-2	Forward	GGCAACCCCATCAAGAGGATTCAAT
	Reverse	CACACTGCTGAGGAGGGG
α-SMA	Forward	TTCGTGACTACTGCTGAGCGTGAGA
	Reverse	AAAGATGGCTGGAAGAGGGTC
Bcl-2	Forward	TCCTTCCAGCCTGAGAGCAAC
	Reverse	GCGACGGTAGCGACGAGAG
Bax	Forward	ATGGGCTGGACACTGGACTTC
	Reverse	GAGTGAGGCAGTGAGGAC

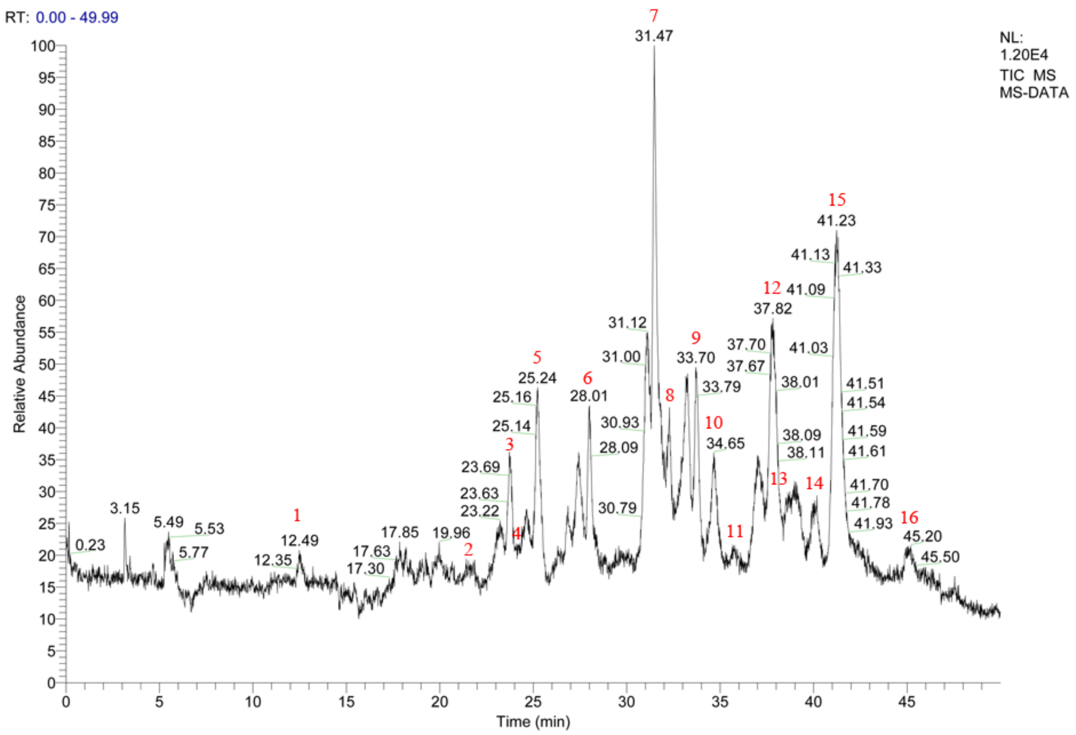

Supplemental Figure 1. The total ion current (TIC) output from the ESI-MS in negative mode.

1

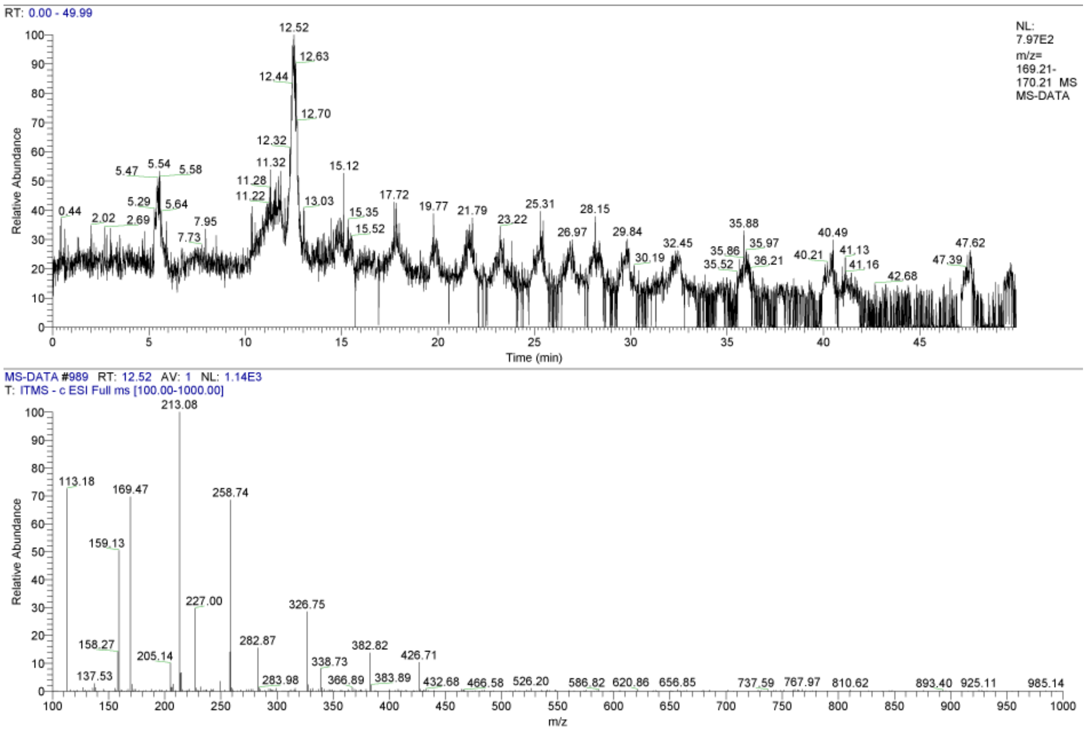
RT: 0.00 - 49.99
NL: 7.97E2 m/z= 169.21-170.21 MS MS-DATA
Relative Abundance
Time (min)
12.52
12.63
12.44
12.70
12.32
11.32
11.28
11.22
5.47 5.54 5.58
5.29 5.64
15.12
13.03
15.35
15.52
17.72
19.77
21.79
23.22
25.31
26.97
28.15
29.84
30.19
32.45
35.88
35.86 35.97
35.52 36.21
40.49
40.21 41.13
41.16
42.68
47.62
47.39
0.44 2.02 2.69 7.95 7.73
MS-DATA #989 RT: 12.52 AV: 1 NL: 1.14E3
T: ITMS - c ESI Full ms [100.00-1000.00]
213.08
113.18
169.47
258.74
159.13
227.00
326.75
158.27
205.14
282.87
382.82
426.71
338.73
137.53 283.98 366.89 383.89 432.68 466.58 526.20 586.82 620.86 656.85 737.59 767.97 810.62 893.40 925.11 985.14
m/z

2

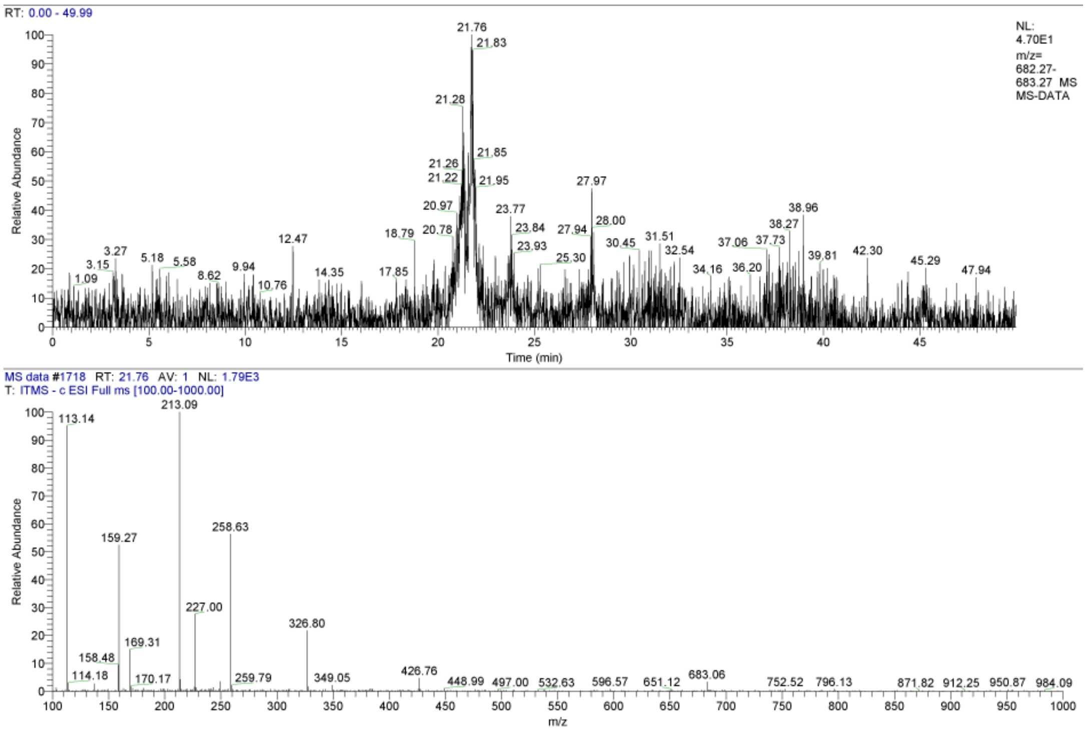
RT: 0.00 - 49.99
NL: 4.70E1 m/z= 682.27-683.27 MS MS-DATA
Relative Abundance
Time (min)
21.76
21.83
21.28
21.26 21.85
21.22 21.95
20.97
20.78
27.97
23.77
23.84 27.94 28.00
23.93
25.30
30.45 31.51
32.54
38.96
38.27
37.06 37.73
39.81
42.30
45.29
47.94
34.16 36.20
18.79
17.85
12.47
14.35
3.27 5.18 5.58
3.15 8.62 9.94
1.09 10.76
MS data #1718 RT: 21.76 AV: 1 NL: 1.79E3
T: ITMS - c ESI Full ms [100.00-1000.00]
213.09
113.14
159.27
258.63
227.00
326.80
169.31
158.48
114.18 170.17 259.79 349.05 426.76 448.99 497.00 532.63 596.57 651.12 683.06 752.52 796.13 871.82 912.25 950.87 984.09
m/z

3

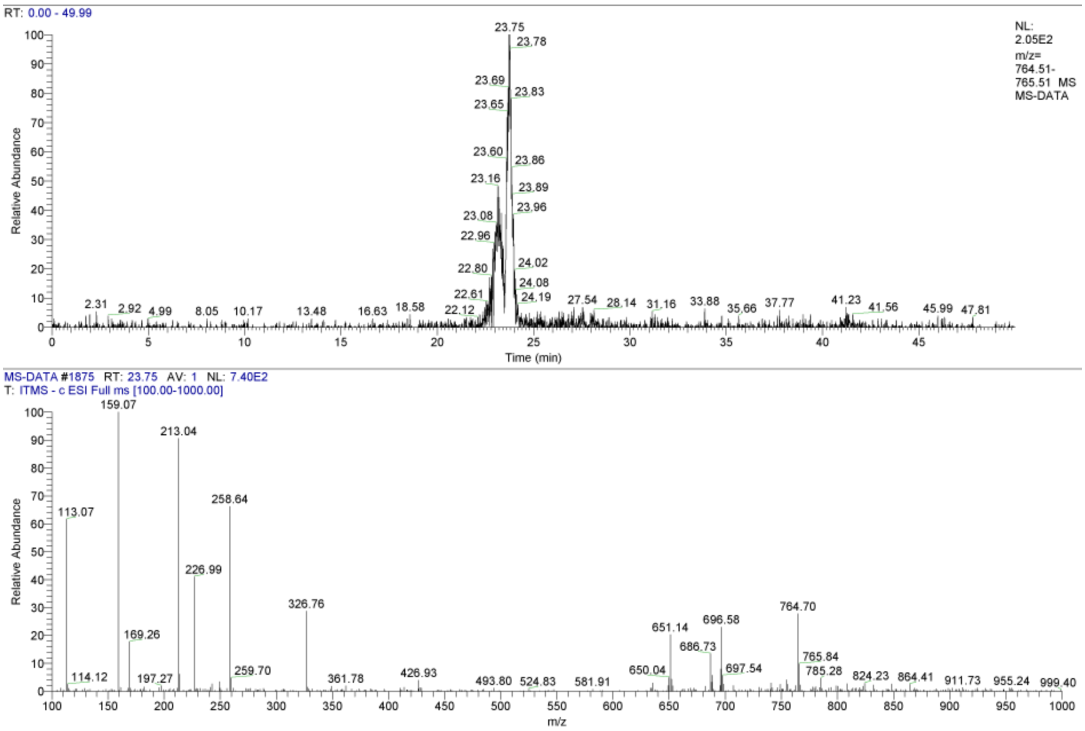
RT: 0.00 - 49.99
NL: 2.05E2 m/z= 764.51-765.51 MS MS-DATA
Relative Abundance
Time (min)
MS-DATA #1875 RT: 23.75 AV: 1 NL: 7.40E2
T: ITMS - c ESI Full ms [100.00-1000.00]
m/z

4

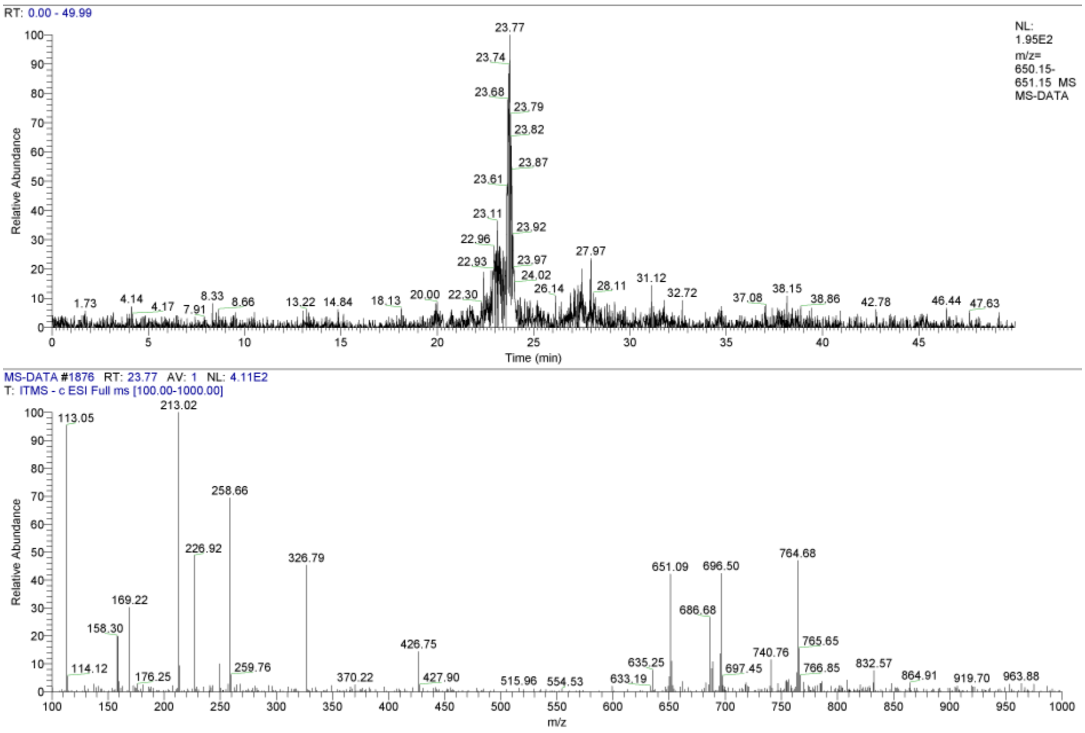
RT: 0.00 - 49.99
NL: 1.95E2 m/z= 650.15-651.15 MS MS-DATA
Relative Abundance
Time (min)
MS-DATA #1876 RT: 23.77 AV: 1 NL: 4.11E2
T: ITMS - c ESI Full ms [100.00-1000.00]
m/z

5

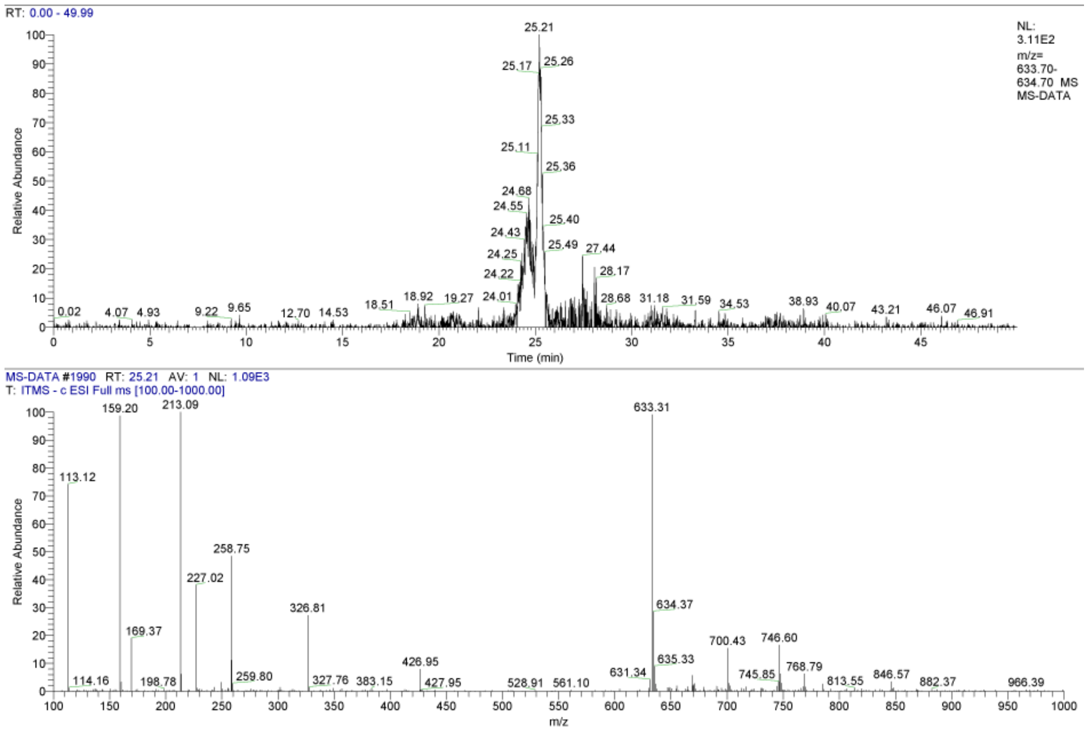
RT: 0.00 - 49.99
NL: 3.11E2 m/z= 633.70-634.70 MS MS-DATA
Relative Abundance
Time (min)
25.21
25.17 25.26
25.33
25.11
25.36
24.68
24.55
25.40
24.43
25.49 27.44
24.25
24.22 28.17
0.02 4.07 4.93 9.22 9.65 12.70 14.53 18.51 18.92 19.27 24.01 28.68 31.18 31.59 34.53 38.93 40.07 43.21 46.07 46.91
MS-DATA #1990 RT: 25.21 AV: 1 NL: 1.09E3
T: ITMS - c ESI Full ms [100.00-1000.00]
Relative Abundance
m/z
159.20 213.09 633.31
113.12
258.75
227.02
326.81 634.37
169.37 700.43 746.60
426.95 635.33 768.79
114.16 198.78 259.80 327.76 383.15 427.95 528.91 561.10 631.34 745.85 813.55 846.57 882.37 966.39

6

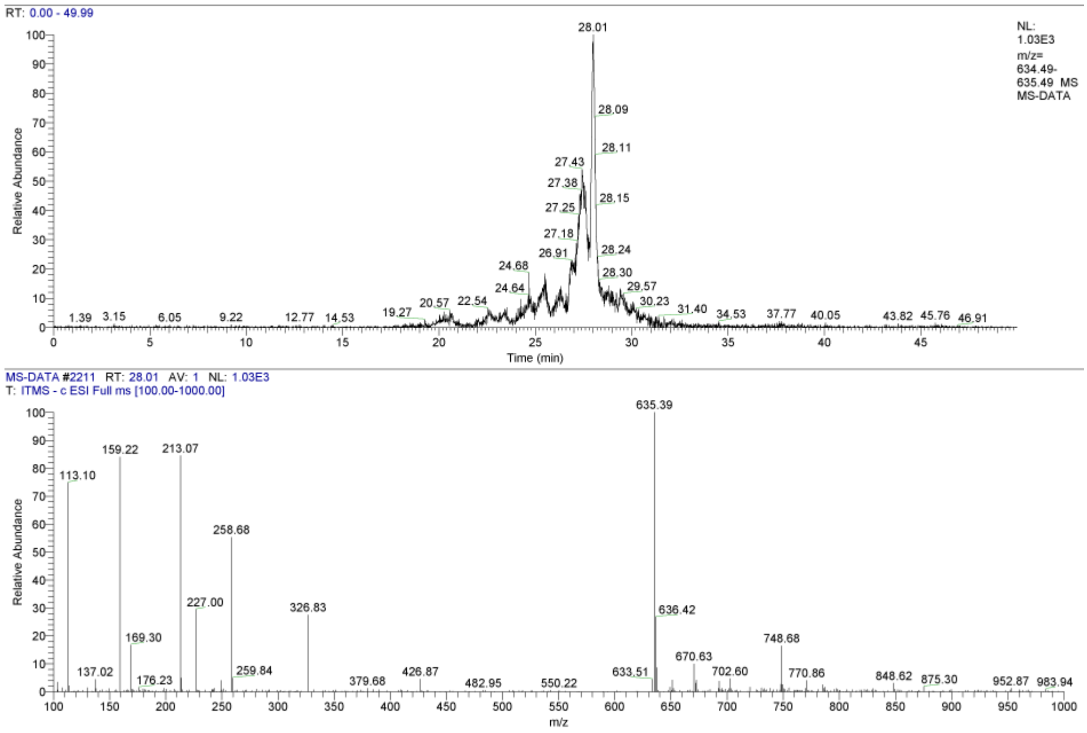
RT: 0.00 - 49.99
NL: 1.03E3 m/z= 634.49-635.49 MS MS-DATA
Relative Abundance
Time (min)
28.01
28.09
28.11
27.43
27.38
27.25 28.15
27.18
26.91 28.24
24.68
28.30
24.64 29.57
1.39 3.15 6.05 9.22 12.77 14.53 19.27 20.57 22.54 30.23 31.40 34.53 37.77 40.05 43.82 45.76 46.91
MS-DATA #2211 RT: 28.01 AV: 1 NL: 1.03E3
T: ITMS - c ESI Full ms [100.00-1000.00]
Relative Abundance
m/z
635.39
159.22 213.07
113.10
258.68
227.00 326.83 636.42
169.30 748.68
670.63
137.02 176.23 259.84 379.68 426.87 482.95 550.22 633.51 702.60 770.86 848.62 875.30 952.87 983.94

7

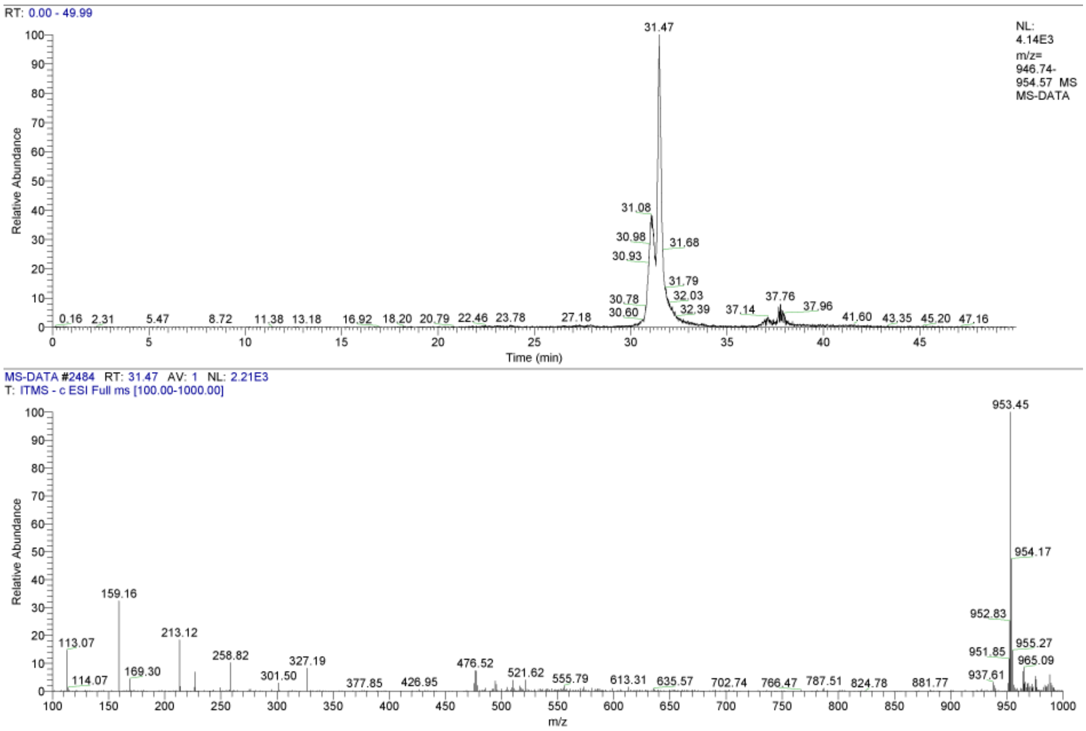
RT: 0.00 - 49.99
NL: 4.14E3 m/z= 946.74-954.57 MS MS-DATA
31.47
31.08
30.98
31.68
30.93
31.79
32.03
32.39
30.78
30.60
37.76
37.96
37.14
0.16 2.31 5.47 8.72 11.38 13.18 16.92 18.20 20.79 22.46 23.78 27.18 41.60 43.35 45.20 47.16
Relative Abundance
Time (min)
MS-DATA #2484 RT: 31.47 AV: 1 NL: 2.21E3
T: ITMS - c ESI Full ms [100.00-1000.00]
953.45
954.17
159.16
952.83
213.12
113.07
258.82
327.19
951.85
955.27
965.09
476.52
521.62
937.61
114.07 169.30 301.50 377.85 426.95 555.79 613.31 635.57 702.74 766.47 787.51 824.78 881.77
Relative Abundance
m/z

8

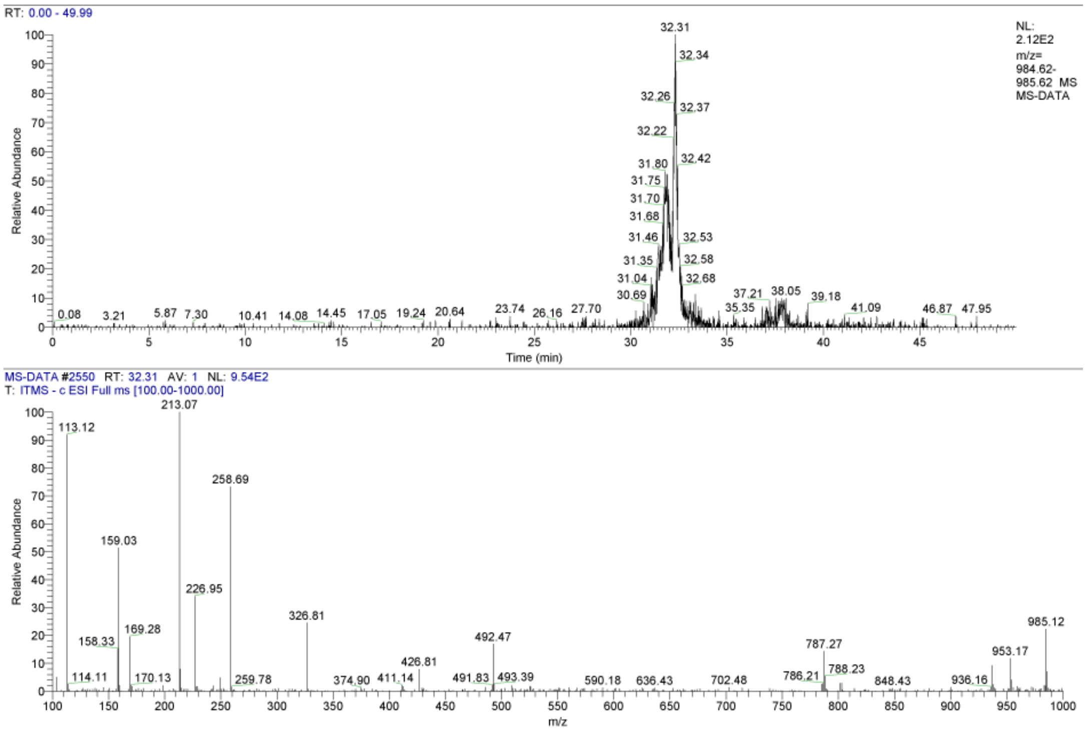
RT: 0.00 - 49.99
NL: 2.12E2 m/z= 984.62-985.62 MS MS-DATA
32.31
32.34
32.26
32.37
32.22
31.80
32.42
31.75
31.70
31.68
31.46
32.53
31.35
32.58
31.04
32.68
30.69
37.21
38.05
39.18
35.35
41.09
46.87 47.95
0.08 3.21 5.87 7.30 10.41 14.08 14.45 17.05 19.24 20.64 23.74 26.16 27.70
Relative Abundance
Time (min)
MS-DATA #2550 RT: 32.31 AV: 1 NL: 9.54E2
T: ITMS - c ESI Full ms [100.00-1000.00]
213.07
113.12
258.69
159.03
226.95
326.81
169.28
158.33
492.47
985.12
787.27
953.17
426.81
788.23
114.11 170.13 259.78 374.90 411.14 491.83 493.39 590.18 636.43 702.48 786.21 848.43 936.16
Relative Abundance
m/z

9

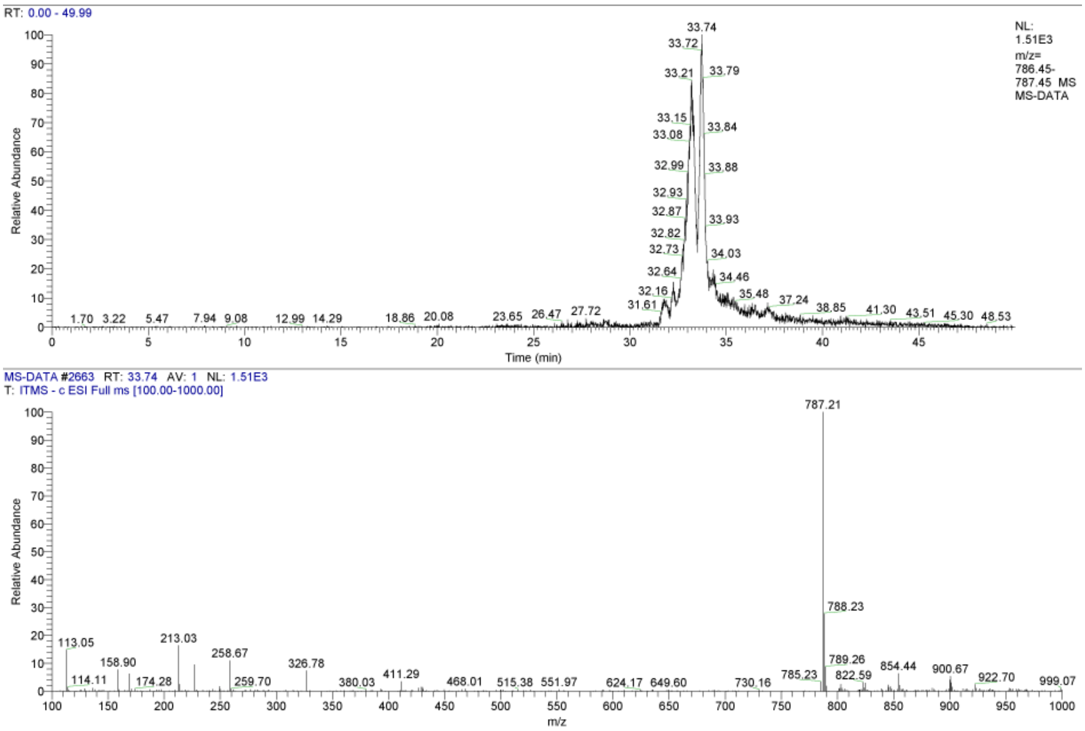
RT: 0.00 - 49.99
NL:
1.51E3
m/z=
786.45-
787.45 MS
MS-DATA
Relative Abundance
Time (min)
33.74
33.72
33.21
33.79
33.15
33.08
33.84
32.99
33.88
32.93
32.87
33.93
32.82
32.73
34.03
32.64
34.46
32.16
35.48
31.61
37.24
38.85
41.30
43.51
45.30
48.53
1.70 3.22 5.47 7.94 9.08 12.99 14.29 18.86 20.08 23.65 26.47 27.72
MS-DATA #2663 RT: 33.74 AV: 1 NL: 1.51E3
T: ITMS - c ESI Full ms [100.00-1000.00]
m/z
787.21
788.23
113.05
213.03
258.67
158.90
326.78
114.11
174.28
259.70
380.03
411.29
468.01
515.38
551.97
624.17
649.60
730.16
785.23
789.26
822.59
854.44
900.67
922.70
999.07

10

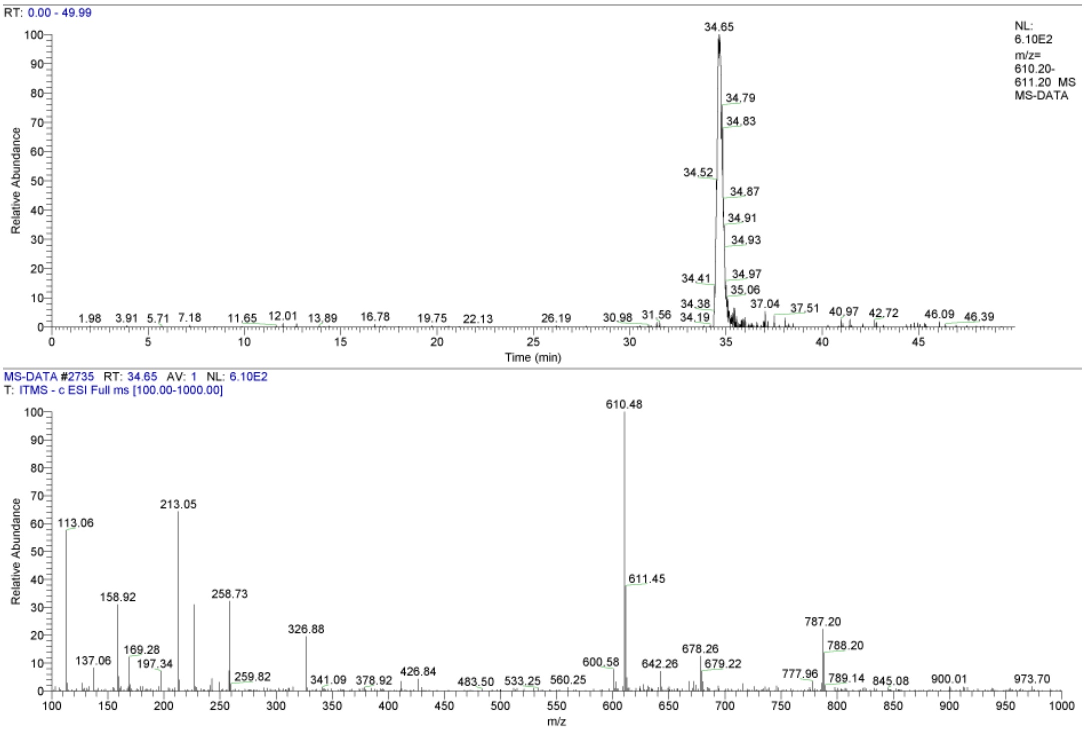
RT: 0.00 - 49.99
NL:
6.10E2
m/z=
610.20-
611.20 MS
MS-DATA
Relative Abundance
Time (min)
34.65
34.79
34.83
34.52
34.87
34.91
34.93
34.41
34.97
34.38
35.06
34.19
37.04
37.51
40.97
42.72
46.09
46.39
1.98 3.91 5.71 7.18 11.65 12.01 13.89 16.78 19.75 22.13 26.19 30.98 31.56
MS-DATA #2735 RT: 34.65 AV: 1 NL: 6.10E2
T: ITMS - c ESI Full ms [100.00-1000.00]
m/z
610.48
213.05
113.06
611.45
158.92
258.73
326.88
787.20
169.28
788.20
137.06
197.34
678.26
600.58
642.26
679.22
259.82
341.09
378.92
426.84
483.50
533.25
560.25
777.96
789.14
845.08
900.01
973.70

11

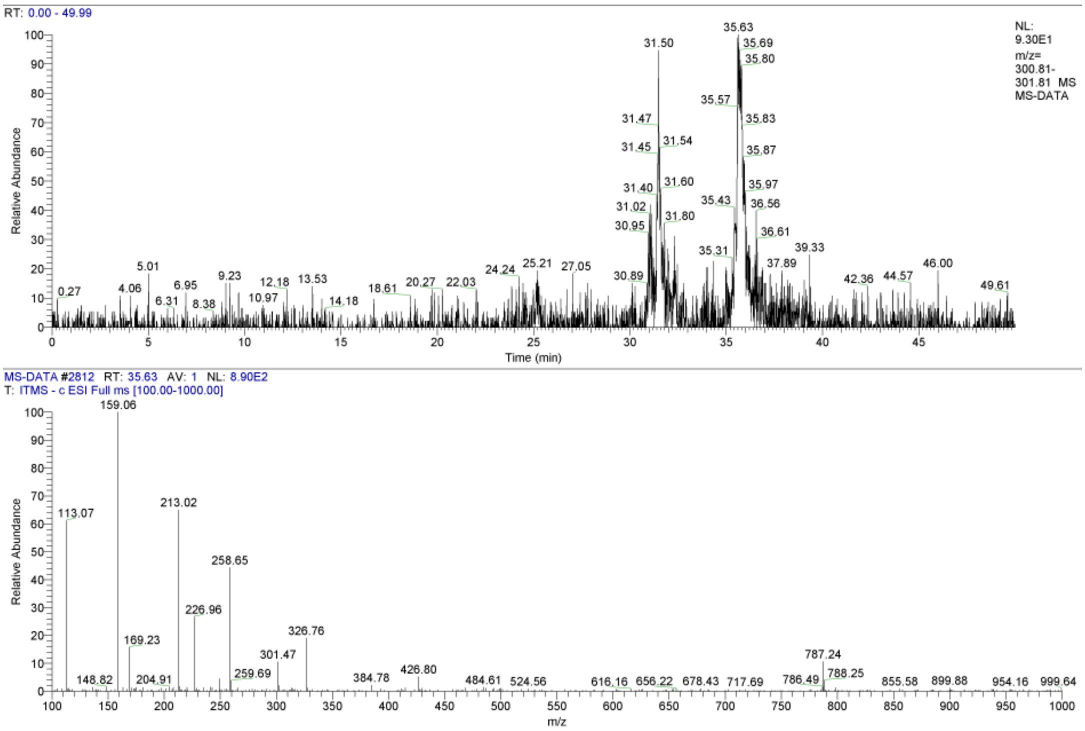
RT: 0.00 - 49.99
NL: 9.30E1 m/z= 300.81-301.81 MS MS-DATA
Relative Abundance
Time (min)
35.63
31.50
MS-DATA #2812 RT: 35.63 AV: 1 NL: 8.90E2
T: ITMS - c ESI Full ms [100.00-1000.00]
Relative Abundance
m/z
159.06
113.07
213.02
258.65
226.96
169.23
326.76
301.47
426.80
787.24

12

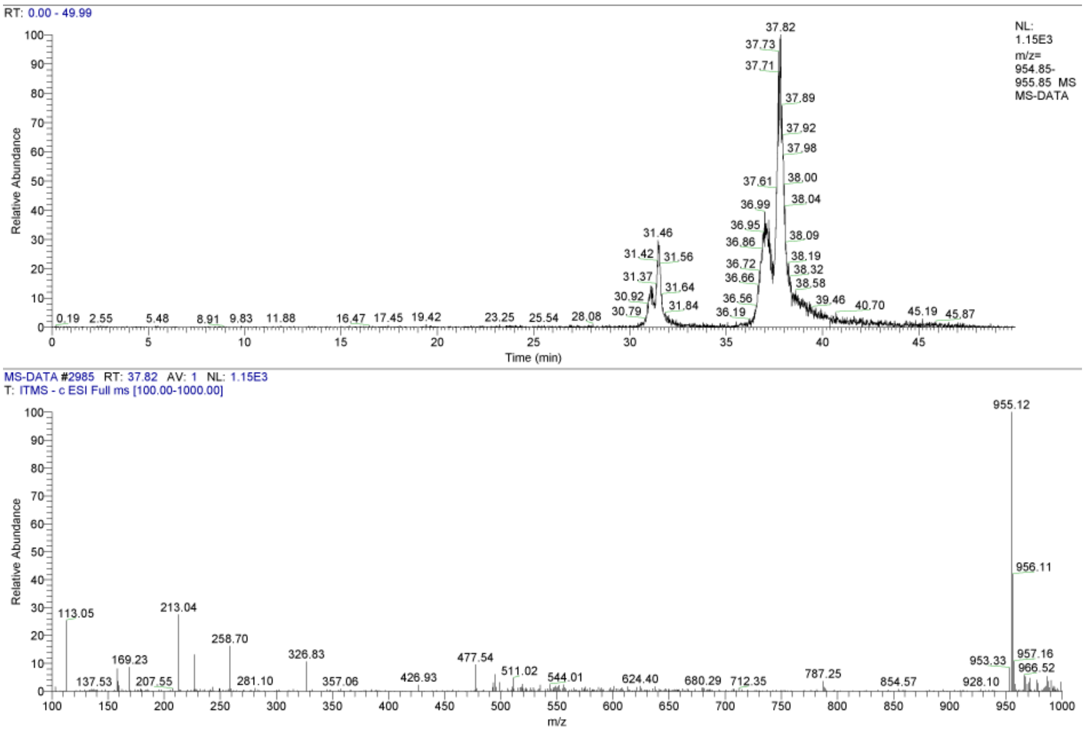
RT: 0.00 - 49.99
NL: 1.15E3 m/z= 954.85-955.85 MS MS-DATA
Relative Abundance
Time (min)
37.82
31.46
MS-DATA #2985 RT: 37.82 AV: 1 NL: 1.15E3
T: ITMS - c ESI Full ms [100.00-1000.00]
Relative Abundance
m/z
955.12
956.11
113.05
213.04
258.70
169.23
326.83
477.54
787.25

13

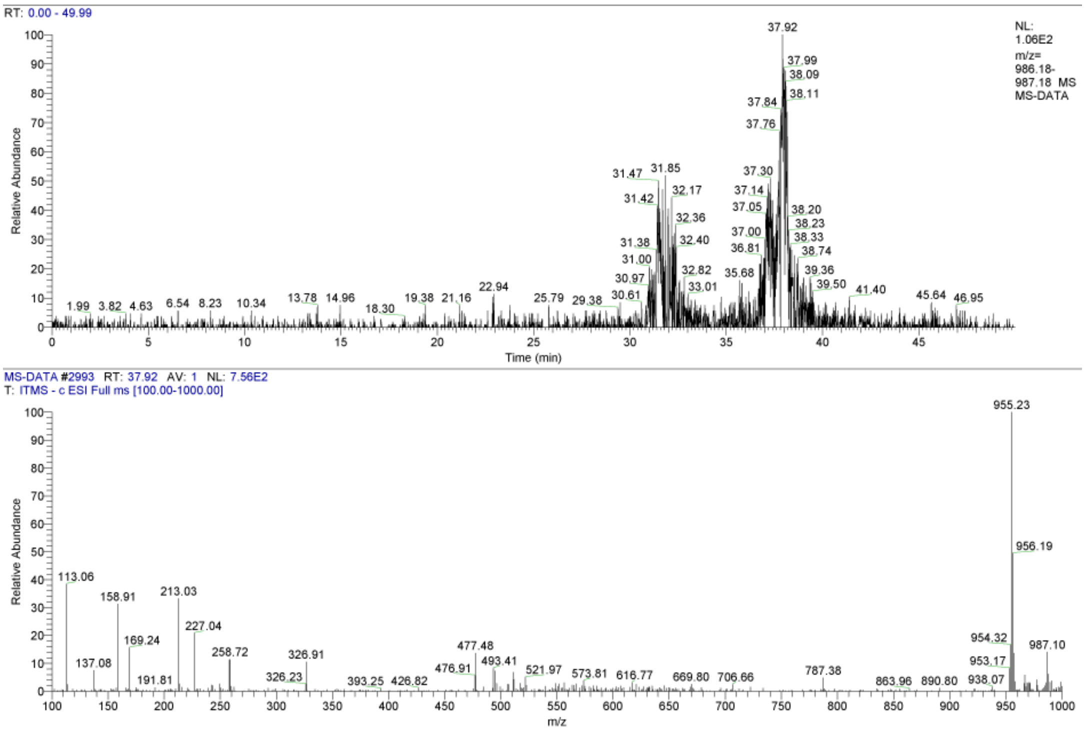
RT: 0.00 - 49.99
NL: 1.06E2 m/z= 986.18-987.18 MS MS-DATA
Relative Abundance
Time (min)
37.92
31.85
31.47
MS-DATA #2993 RT: 37.92 AV: 1 NL: 7.56E2
T: ITMS - c ESI Full ms [100.00-1000.00]
955.23
956.19
113.06
158.91
213.03
227.04
987.10
m/z

14

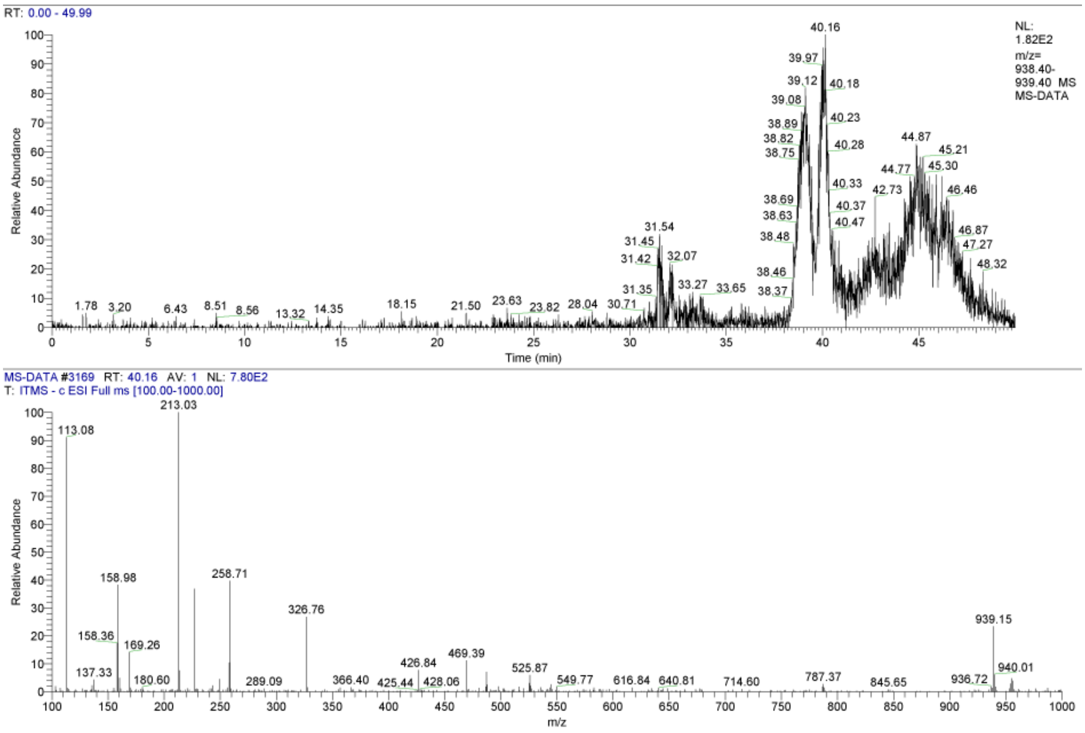
RT: 0.00 - 49.99
NL: 1.82E2 m/z= 938.40-939.40 MS MS-DATA
Relative Abundance
Time (min)
40.16
39.97
44.87
31.54
MS-DATA #3169 RT: 40.16 AV: 1 NL: 7.80E2
T: ITMS - c ESI Full ms [100.00-1000.00]
213.03
113.08
158.98
258.71
326.76
939.15
m/z

15

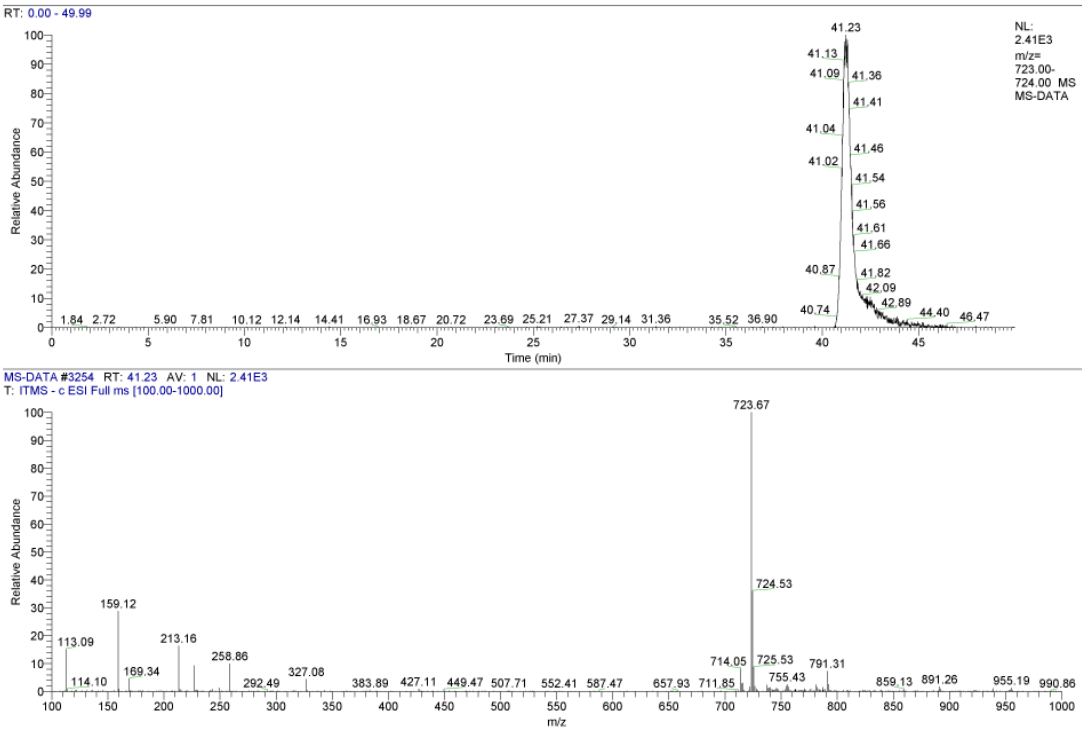

16

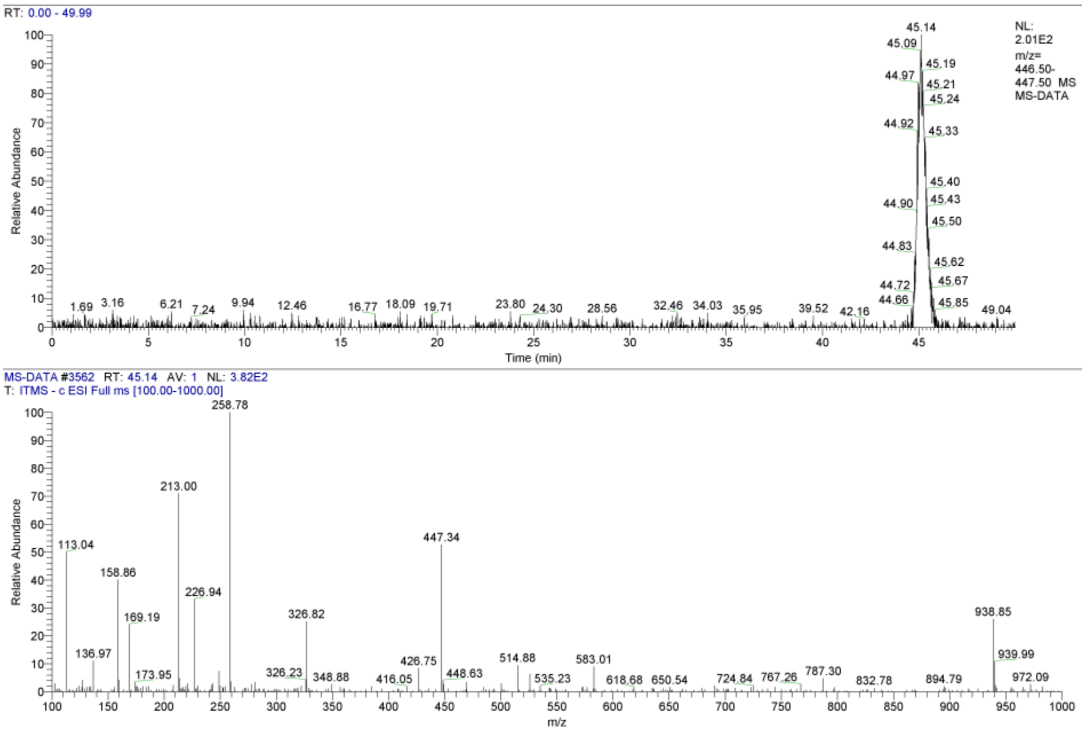

Supplemental Figure 2. MS spectra of the putative 16 compounds in EF. The number of each spectrum corresponds to the peak number in Table 2, Figure 1, Supplemental Figure 1 and Supplemental Figure 3.

1

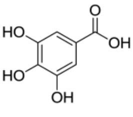

Chemical Formula: $C_7H_6O_5$
Exact Mass: 170.02152

2

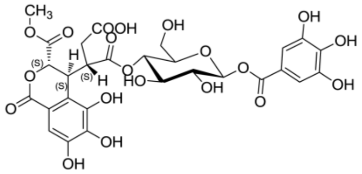

Chemical Formula: $C_{28}H_{28}O_{20}$
Exact Mass: 684.11739

3

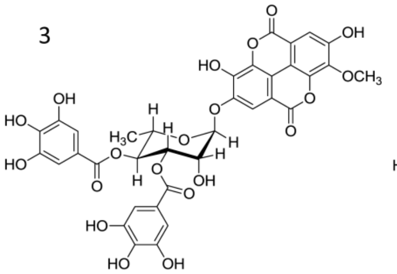

Chemical Formula: $C_{35}H_{26}O_{20}$
Exact Mass: 766.10174

4

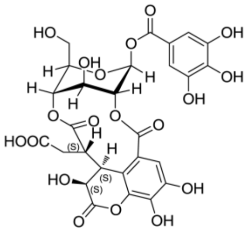

Chemical Formula: $C_{27}H_{24}O_{19}$
Exact Mass: 652.09118

5

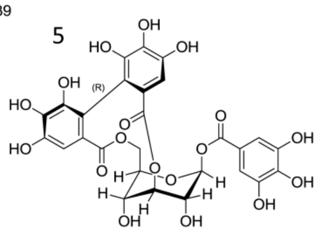

Chemical Formula: $C_{27}H_{22}O_{18}$
Exact Mass: 634.08061

6

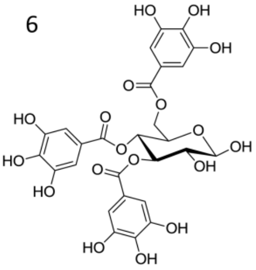

Chemical Formula: $C_{27}H_{24}O_{18}$
Exact Mass: 636.09626

7

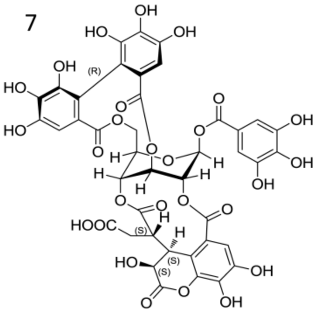

Chemical Formula: $C_{41}H_{30}O_{27}$
Exact Mass: 954.09745

8

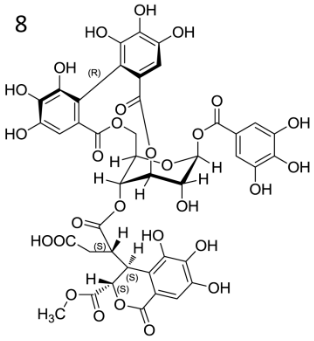

Chemical Formula: $C_{42}H_{34}O_{28}$
Exact Mass: 986.12366

9

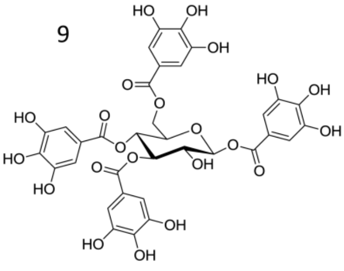

Chemical Formula: $C_{34}H_{28}O_{22}$
Exact Mass: 788.10722

11

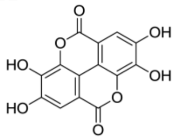

Chemical Formula: $C_{14}H_6O_8$
Exact Mass: 302.00627

12

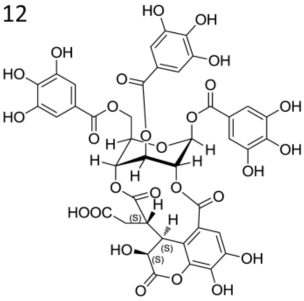

Chemical Formula: $C_{41}H_{32}O_{27}$
Exact Mass: 956.11310

13

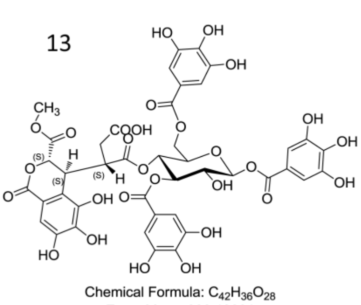

Chemical Formula: $C_{42}H_{36}O_{28}$
Exact Mass: 988.13931

14

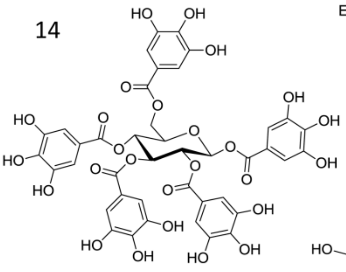

Chemical Formula: $C_{41}H_{32}O_{26}$
Exact Mass: 940.11818

16

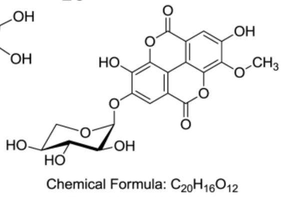

Chemical Formula: $C_{20}H_{16}O_{12}$
Exact Mass: 448.06418

Supplemental Figure 3. Structures of the 14 phenolic compounds identified from EF based on the HPLC-MS data and the known structures of reference compounds. Compound numbers, chemical formula and exact mass are presented.

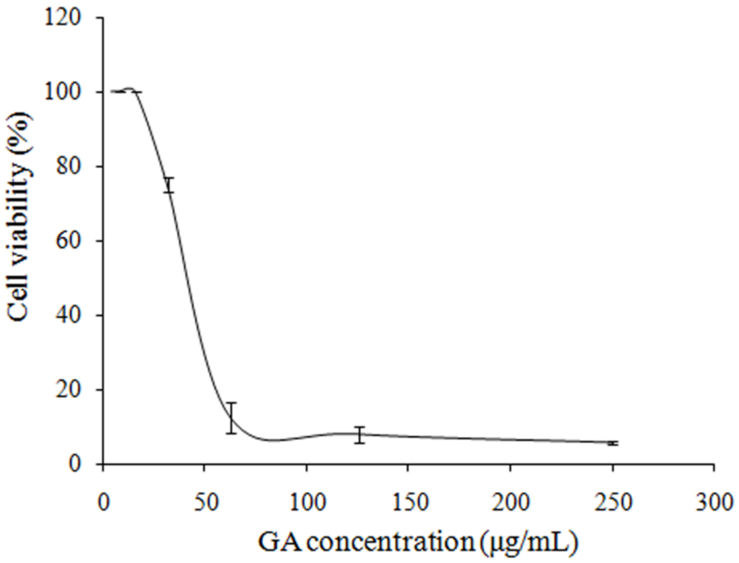

Supplemental Figure 4. Effect of GA on HSC-T6 cell viability. The viability rate (%) of HSC-T6 cells manifest decreased with increased GA concentration.
